# Cancer resistance via the downregulation of the tumor suppressors *RKIP* and *PTEN* expressions: therapeutic implications

**DOI:** 10.37349/etat.2023.00128

**Published:** 2023-04-20

**Authors:** Matthew Moghaddam, Silvia Vivarelli, Luca Falzone, Massimo Libra, Benjamin Bonavida

**Affiliations:** 1 Department of Microbiology, Immunology and Molecular Genetics, David Geffen School of Medicine, Jonsson Comprehensive Cancer Center, University of California, Los Angeles (UCLA), East Los Angeles, CA 90095, USA; 2 Department of Biomedical and Dental Sciences and Morphofunctional Imaging, Occupational Medicine Section, University of Messina, 98125 Messina, Italy; 3 Epidemiology and Biostatistics Unit, National Cancer Institute IRCCS Fondazione G. Pascale, 80131 Naples, Italy; 4 Department of Biomedical and Biotechnological Sciences, University of Catania, 95123 Catania, Italy; 5 Research Centre for Prevention, Diagnosis and Treatment of Cancer, University of Catania, 95123 Catania, Italy; Université Paris-Saclay, France

**Keywords:** RKIP, PTEN, cross-talks, signaling, bioinformatics, resistance

## Abstract

The Raf kinase inhibitor protein (RKIP) has been reported to be underexpressed in many cancers and plays a role in the regulation of tumor cells' survival, proliferation, invasion, and metastasis, hence, a tumor suppressor. RKIP also regulates tumor cell resistance to cytotoxic drugs/cells. Likewise, the tumor suppressor, phosphatase and tensin homolog (PTEN), which inhibits the phosphatidylinositol 3 kinase (PI3K)/AKT pathway, is either mutated, underexpressed, or deleted in many cancers and shares with RKIP its anti-tumor properties and its regulation in resistance. The transcriptional and posttranscriptional regulations of RKIP and PTEN expressions and their roles in resistance were reviewed. The underlying mechanism of the interrelationship between the signaling expressions of RKIP and PTEN in cancer is not clear. Several pathways are regulated by RKIP and PTEN and the transcriptional and post-transcriptional regulations of RKIP and PTEN is significantly altered in cancers. In addition, RKIP and PTEN play a key role in the regulation of tumor cells response to chemotherapy and immunotherapy. In addition, molecular and bioinformatic data revealed crosstalk signaling networks that regulate the expressions of both *RKIP* and *PTEN*. These crosstalks involved the mitogen-activated protein kinase (MAPK)/PI3K pathways and the dysregulated nuclear factor-kappaB (NF-κB)/Snail/Yin Yang 1 (YY1)/RKIP/PTEN loop in many cancers. Furthermore, further bioinformatic analyses were performed to investigate the correlations (positive or negative) and the prognostic significance of the expressions of *RKIP* or *PTEN* in 31 different human cancers. These analyses were not uniform and only revealed that there was a positive correlation between the expression of *RKIP* and *PTEN* only in few cancers. These findings demonstrated the existence of signaling cross-talks between RKIP and PTEN and both regulate resistance. Targeting either RKIP or PTEN (alone or in combination with other therapies) may be sufficient to therapeutically inhibit tumor growth and reverse the tumor resistance to cytotoxic therapies.

## Introduction

Normal human cells express several tumor suppressor gene products to protect them from neoplastic transformation and the induction of cancer [1, 2]. Most cancers express low levels of the tumor suppressor Raf kinase inhibitor protein (*RKIP*) [3] and low levels of the tumor suppressor phosphatase and tensin homolog (*PTEN*) deleted on chromosome 10 or mutated [4]. The expression of these gene products in various cancers has been reported to inhibit cell proliferation and cell survival, inhibit metastases, and respond to cytotoxic/apoptotic stimuli [5, 6].

We have reported that many cancers exhibit a dysregulated nuclear factor-kappaB (NF-κB)/Snail/Yin Yang 1 (YY1)/RKIP/PTEN loop that primarily is responsible for the phenotypic properties of cancer cells, namely, cell proliferation and viability, migration and metastasis, and resistance to both cytotoxic drugs and to cytotoxic T and natural killer (NK) cells [7, 8]. In this loop, the overexpression and activities of NF-κB, Snail, and YY1 regulate the inhibition of *RKIP* and *PTEN* expressions. In contrast, the inhibition of either of the gene products, *NF-κB*, *Snail*, or *YY1* resulted in the upregulation of the expressions and activities of both RKIP and PTEN [5, 9–11]. Further, Snail is a transcriptional repressor of *RKIP* [12] and YY1 is a transcriptional activator of *Snail* [13] and a repressor of *PTEN* [5, 14]. In the dysregulated loop, either one of the gene products, directly or indirectly, regulates other gene products in the loop. Thus, *RKIP* and *PTEN* regulate each other indirectly.

We hypothesized that, in addition to the indirect regulation between RKIP and PTEN, there may exist a direct regulation via crosstalk signaling pathways. In this review, we have reviewed the general properties and the transcriptional and post-transcriptional regulations of RKIP and PTEN in cancer and their roles in the regulation of both chemo- and immune-resistance, the various cross-talk signaling pathways shared by RKIP and PTEN, bioinformatic analyses exploring the cross-talk pathways between *RKIP* and *PTEN* gene products, and the therapeutic implications of these new findings.

## RKIP characteristics

### General properties

Genes are the basic unit of heredity for all individuals and can act as a set of instructions to inform cells how to grow and what to do [15]. There are several thousand different types of genes in the human body, approximately 20,000 many of which can code to make proteins [16]. RKIP is an inhibitory protein that was first discovered by Yeung et al. [17]. RKIP was initially identified as a protein that binds phospholipids in the metabolism of lipids [18]. Thus, RKIP was previously called phosphatidylethanolamine binding protein 2 (PEBP2) [19]. In 1999, Yeung et al. [17] identified that this protein inhibits Raf's ability to phosphorylate MEK, earning the name RKIP. The role of RKIP was shown in a yeast two-hybrid experiment to regulate the Raf-MEK-extracellular signal-regulated kinase (ERK) signaling pathway by binding to the Raf-1 isoform and interfering with MEK phosphorylation [17].

In humans, RKIP messenger RNA (mRNA) is 1,434 base pairs (bp) long, and by belonging to the PEBP family, it does not have a significant sequence homology with other proteins of known structure or function [20]. The *RKIP* gene is located on chromosome 12q24.23 and consists of four exons that are spread across approximately 10 kilo base (kb) pairs [21]. The molecular weight of RKIP is 21,057 Da [22]. The RKIP transcription start site (TSS) is 146 bp upstream from the ATG site. Cap analysis gene expression (CAGE) not only helped researchers identify the TSS but also helped researchers to notice that most of the TSS of RKIP are in groups from 21 bases to 99 bases downstream of the RefSeq TSS [23].

There is a 95% similarity between human RKIP and bovine RKIP and an 85.5% similarity to rat mRNA [21]. This is significant because the human *RKIP* mRNA encodes a 187 amino acid protein that shares a 186 amino acid overlap with the bovine 21 kDa RKIP and a 187 amino acid overlap with rat 23 kDa RKIP [24]. Although RKIP amino acid sequences amongst humans, bovines, and rats are quite similar, the RKIP pocket may play different roles for different species at different pH levels. Previous studies agree that nucleotide-binding of RKIP is enhanced at lower pH values [25]. However, one study discovered that at neutral pH levels, which is approximately at normal physiological conditions, rat recombinant RKIP failed to bind to nucleotides [26]. Yet, when human RKIP was used, it was revealed that the RKIP ligand pocket will bind various nucleotides when physiological conditions are similar [25].

Mammals contain three Raf protein isoforms, A-Raf, B-Raf, and C-Raf (also known as Raf-1), which originated from 3 independent genes. Raf-1 was the first to be identified, and amongst all three isoforms, it was the principal focus of attention for twenty years [27]. Raf family proteins have similar but independent cellular functions. All Raf proteins share MEK ½ kinases as substrates, which activate ERK ½ signaling. RKIP is not a substrate of either Raf-1 or MEK, but it interferes with the interaction between them [28].

*RKIP* has been reported to be a metastasis suppressor gene product [5, 29] and also regulates tumor cell resistance to both chemotherapy and immunotherapy [30]. In addition, RKIP was also reported to inhibit epithelial-mesenchymal transition (EMT) [31]. Metastasis is a major cause of cancer mortality because the process involves primary tumors spreading to secondary organs [32]. The process of metastasis involves tumor cells disseminating from the primary tumor, passing through the basement membrane, persevering in the circulatory system, and invading the secondary site [33]. Metastasis suppressor genes inhibit the development of metastasis without blocking primary tumor growth. RKIP's role as a metastasis suppressor was initially investigated by Fu et al. [29] comparing the metastatic prostate cancer cell line (c4-2B) to the non-metastatic cell line (LNCaP). Through immunohistochemistry (IHC), their results showed that *RKIP* expression was associated with the suppression of prostate cancer metastasis while the loss of *RKIP* expression was associated with metastasis [29].

### RKIP expression in cancers

As mentioned earlier, low levels of *RKIP* are associated with a high incidence of tumor growth and metastasis in cancer patients [34, 35]. While invasion and intravasation are rather early events in the metastatic cascade, it is important to determine whether RKIP is able to support late metastatic events such as colonization and growth at a distant site [36]. Therefore, in a study by Dangi-Garimella et al. [36], researchers injected luciferase-labeled 1,833 cells with wild-type (wt) RKIP into the left cardiac ventricle of mice in order to bypass the intravasation step. Their results illustrated a marked decrease in bone metastasis, confirming that RKIP is in fact a suppressor of breast cancer metastasis [36].

Multiple studies have shown that a loss or reduction in *RKIP* expression is frequently found in many solid tumor cancers including breast, melanoma, and prostate [37]. Hagan et al. [38] were the first to identify that, in human breast cancer, RKIP must be downregulated for metastasis to develop. One study by Al-Mulla et al. [39] discovered that patients with breast cancer exhibited larger-sized tumors and a higher tumor grade when RKIP was lost or reduced. Additionally, Al-Mulla et al. [39] analyzed a publicly available breast cancer gene expression microarray data set from 115 women published previously. They discovered that RKIP mRNA levels were significantly lower in metastatic breast cancer patients compared to those that were non-metastatic [39].

Similarly, another study by Penas et al. [40] used IHC to determine RKIP expression in human melanocytic lesions. Taken from 239 melanoma and 75 nevi samples, Penas et al. [40] found that RKIP staining in the melanoma samples exhibited an overall decrease compared to benign lesions. Therefore, they concluded that both RKIP mRNA and protein expressions were significantly lower in melanoma cells compared to primary cultures of melanocytes. These results confirmed that RKIP loss is associated with melanoma dissemination [40].

### Crystal structure

RKIP tends to crystallize in two asymmetric molecules (180 and 185 residues). The closely packed structure of RKIP is based on nine beta-strands and four alpha-helices, and together they are folded in a unique pattern to provide stability [41–43]. The tertiary structure of RKIP is globular-like and contains a hydrophobic cavity, which serves as the ligand-binding pocket [23]. The ligand-binding pocket of RKIP is comprised of 16 amino acid residues and it regulates the binding of several nucleotides including guanosine triphosphate (GTP), guanosine diphosphate (GDP) [41], and flavin mononucleotide (FMN) [25]. GTP is a nucleoside phosphate composed of a ribonucleoside and three phosphate groups while the removal of two phosphate groups yields GDP. The function of GTPs is to use the energy stored in their phosphate bonds for metabolic processes and protein synthesis. The release of energy from GTP after it becomes hydrolyzed results in the enzyme becoming GDP. Many phospholipids and non-lipid organic compounds, like locostatin, are also accommodated by the RKIP ligand-binding pocket. The ligand-binding pocket's role is to inhibit the ERK pathway and interact with the phosphorylated N-region of Raf-1 [23].

### RKIP-mediated signaling

The Raf-MEK-ERK signaling pathway plays a critical role in the control of cell proliferation, differentiation, migration, and apoptosis. The pathway begins when the activated receptor tyrosine kinases (RTKs) bind with the guanine nucleotide exchange factor, SOS. Afterward, RAS becomes activated as GDP gets replaced for GTP, which activates Raf. Active Raf phosphorylates MEK, which activates ERK. The attachment of RKIP to Raf inhibits the phosphorylation of MEK. This, in turn, negatively regulates the flow of signals down the Raf-MEK, ERK pathway [27]. RKIP is also able to bind to MEK, and to a lesser extent ERK. When RKIP binds to the kinase domain of Raf-1, it prevents its phosphorylation by PAK and Src kinases at Ser338 and Tyr340/341 [43, 44]. By blocking the Raf-1-MEK-ERK signaling cascade, RKIP inhibits downstream the AP-1 transcription factor. Through the modulation of the mitogen-activated protein kinase (MAPK) pathway, RKIP encourages a balanced cell cycle kinetics and replication process through differential regulation of various pathways including cell proliferation and apoptosis [45, 46]. In a study by Al-Mulla et al. [39], researchers found that the upregulation of RKIP shortened the nuclear envelop breakdown (NEB) to anaphase time, and the downregulation of RKIP accelerates the time from NEB to anaphase. Furthermore, RKIP depletion induces the expression of NEK6 (a molecule known to enhance G2/M transition) while simultaneously down-regulating G2/M checkpoint molecules like Aurora B, cyclin G1, and sirtuin [46]. Their results suggested that subtle changes in cell cycle kinetics may be fundamental to RKIP's role as a metastasis suppressor [46].

Furthermore, RKIP inhibits NF-κB activation by blocking the IkappaB (IκB) phosphorylation by a family of IκB kinase (IKK) kinases. NF-κB is found in nearly all cell types and is involved in the activation of genes in response to the body's detection of infection, inflammation, and other stressful situations that require rapid reprogramming of gene expression [47]. NF-κB is a transcription factor that was first discovered in 1986 as a nuclear factor that binds to the enhancer element of the immunoglobulin kappa light-chain of activated B-cells, thereby receiving the abbreviation NF-κB [48]. NF-κB is normally located in the cytoplasm of non-active cells, but in order for it to function, it must be translocated to the nucleus [47]. IκB is an inhibitory protein that binds with NF-κB in the cytoplasm to keep it in its inactive form [48]. IκB is able to become phosphorylated by active IKK. When IκB becomes phosphorylated, NF-κB becomes active and is then able to translocate to the nucleus where it guides the transcription of several target molecules involved in cell proliferation, apoptosis, and cell migration [49, 50]. Some kinases that activate IKK include NF-κB-inducing kinase (NIK) and transforming growth factor B-activated kinase-1 (TAK-1) [51]. The binding of RKIP to all IKK kinases, including IKK, NIK, and TAK-1, inhibits the activation of the NF-κB cascade.

**Figure 1.**
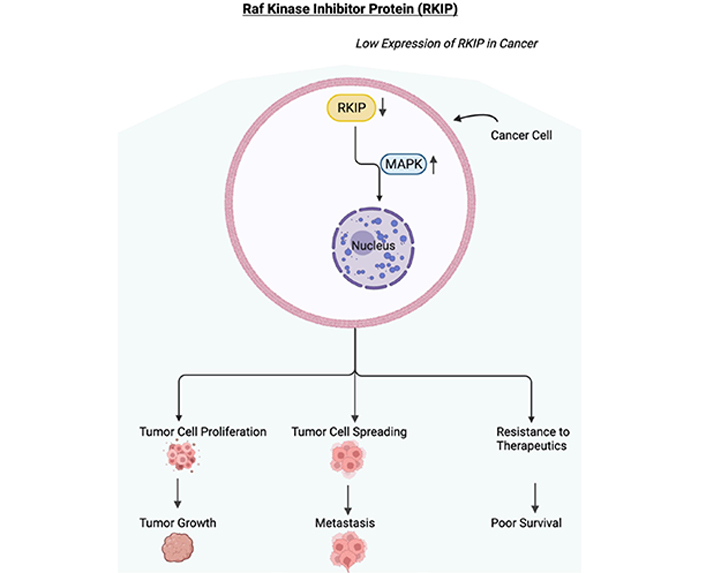
Low expression of RKIP in cancer. Schematic diagram depicting the downregulation or loss of expression of RKIP. In cancer cells, RKIP has been shown to be downregulated or lost resulting in the upregulation of the MAPK pathway. The activated MAPK pathway will initiate the proliferation of tumor cells, the spreading of tumor cells, and resistance to therapeutics. These will result in tumor growth, metastasis, resistance to therapeutics, and in poor survival of the cancer patient. Figure created with BioRender.com

RKIP can become phosphorylated by protein kinase C (PKC), which will release the binding of Raf-1 and the subsequent activation of MEK and ERK (Figure 1) [52]. Phosphorylated RKIP (pRKIP) will bind to and inhibit the G protein coupled-receptor kinase-2 (GRK2) instead of Raf-1, resulting in continued G protein signaling. G proteins are a type of protein that binds to the guanine nucleotides in GTP and GDP [53]. As two RKIP molecules come together, a dimerization is formed, and RKIP displays a higher affinity to GRK2 and a lower affinity to Raf-1 [54]. The RKIP dimer is constructed by disulfide cross-linking (by similarity) RKIP molecules. However, RKIP dimerization and GRK2 binding have been shown to be prevented by a peptide comprising amino acids 127–146 of RKIP [53, 54]. More in detail, the binding of RKIP to GRK2 is defined RKIP^GRK2^ and occurs when RKIP is phosphorylated at S153. RKIP^KIN^ is the intermediary state that enables PKC to phosphorylate RKIP at S153. At this point, pRKIP inhibits GRK2 instead of Raf [54]. Recent evidence from Skinner et al. [55] suggested that RKIP encompasses three discrete states based on its various functional roles. The three different states of RKIP are RKIP^RAF^, RKIP^GRK2^, and RKIP^KIN^. RKIP^RAF^ is the binding of RKIP to Raf when RKIP is not phosphorylated.

### Regulation of *RKIP* expression

#### Transcriptional

The downregulation of *RKIP* expression in multiple types of human cancers is also a result of decreased RKIP transcription. A luciferase assay used on human melanoma A375 and cervical cancer HeLa cells revealed that the full RKIP promoter activity requires the nucleotide region compromising −56 to +261 relative to the TSS [56]. The three kinds of *cis*-acting elements and transcription factors that showed to positively regulate RKIP transcription were specificity protein 1 (Sp1), cyclic adenosine monophosphate (cAMP) response element binding protein (CREB), and the p300 acetylase protein [56].

The binding of Sp1 to a site or sites close to the transcription start showed to mediate transcription assembly when the traditional TATA box was not included [56]. In order to determine the effect of Sp1 on RKIP transcription, researchers synthesized two pairs of oligonucleotides corresponding to two different binding sites and determined their recognition in A375 or HeLa cells by electrophoretic mobility shift assay (EMSA). The first oligonucleotide (Sp1 I) was derived from either the −17 site to −6 site while the second oligonucleotide (Sp1 II) site corresponded to the −5 site to +5 site. The results concluded that the knockdown of Sp1 I and Sp1 II or mutation of these elements decreased RKIP promoter activity [56].

Similarly, the interaction between CREB and transcription factor II B (TFIIB) and TFIID enhances RKIP transcription [56]. When researchers synthesized a pair of oligonucleotides according to CREB binding sites, they noticed that the mutated and deleted CREB binding sites resulted in ∼60% reduction in luciferase activity. Thus, it was concluded that CREB has a positive correlation to RKIP promoter activity [56].

The acetyltransferase p300 increases the rate of RKIP transcription by decondensing the tightly packed chromatin [56]. In order to determine the effect of p300 knockdown or overexpression on *RKIP* promoter activity, researchers transfected A375 cells with p300-specific small interfering RNA (siRNA). This caused a ∼40% reduction in promoter activity. However, when A375 cells were transfected with a p300 expression plasmid, there was a two-fold increase in promoted activity. The results indicated that p300 is one of the major transcription factors that promote RKIP transcription [56].

Androgen receptors (ARs) are another type of transcription factor that directly binds to and regulates RKIP transcription in prostate epithelial cells [57, 58]. In order to determine whether ARs bind to the RKIP promoter, researchers used the androgen hormone, dihydrotestosterone (DHT), on prostate epithelial cells. Through EMSA and chromatin immunoprecipitation (ChIP), it was identified that DHT activates AR, thereby leading to the positive regulation of RKIP [57].

Although there are transcription factors that function as enhancers, there are also transcription factors that inhibit the expression of *RKIP* as well. One type of transcription factor that inhibits *RKIP* expression is Snail. Snail is a zinc finger transcriptional repressor that operates by binding to the E-box *cis*-elements in the RKIP promoter and recruiting mSin3A histone deacetylases containing repressor complexes [12]. One study by Ren et al. [10] used a combination of loss- and gain-of-function approaches and discovered that enhancer of zeste homolog 2 (EZH2) negatively regulated RKIP transcription. Their findings concluded that EZH2 accelerates cancer cell invasion from RKIP inhibition, but this is dependent on the recruitment of Snail to the RKIP promoter [10]. When Snail is present, EZH2 inhibits *RKIP* expression at the transcriptional level, which accelerates cellular invasion [59].

Another type of *RKIP* transcription repressor is the BTB and CNC homology protein 1 (BACH1). BACH1 is a basic leucine zipper transcription factor that was found to enhance the malignancy of breast cancer cells when expression levels were high [60]. Lee et al. [61] demonstrated how the relationship between BACH1 and RKIP exemplifies a double-negative (overall positive) feedback loop by mutually repressing each other's expression. RKIP suppressed BACH1 expression indirectly through signaling, transcriptional, and RNA interference (*let-7*) pathways while BACH1 negatively regulated *RKIP* expression [61]. Another study cloned the BACH1 3′ untranslated region (UTR) containing two *let-7* binding sites into a luciferase assay to confirm whether BACH1 mRNA directly binds *let-7* [62]. The results concluded that BACH1 mRNA is a direct *let-7* target, and RKIP regulates BACH1 via *let-7* binding [62].

#### Epigenetic

Through the screening of cancer cells, researchers have identified a series of regulatory molecules that modulate *RKIP* expression. One type of molecule that plays an important role in the methylation of RKIP histones is the epigenetic silencer EZH2. EZH2 is the catalytic subunit of the multicomponent protein complex called Polycomb repressive complex 2 (PRC2) and is important in epigenetics as it affects the initiation and progression of several diseases [63]. The catalytic activity of EZH2 serves well in the diagnoses and therapies of different pathologies [63]. EZH2 provides instructions for making an enzyme called methyltransferase, which modifies specific histones. By adding a methyl group to histones, histone methyltransferases can suppress the activity of certain genes and influence the type of cell an immature cell will ultimately become (cell fate determination) [64]. EZH2 functions by binding to the proximal E-box of the RKIP promoter, recruiting the suppressor of zeste 12 (Suz12), and inducing the tri-methylation of lysines 9 and 27 of histone 3 (H3-K27) [10, 58]. The methylation of lysine amino acids on histones results in reduced transcription, and expression levels between *RKIP* and *EZH2* were shown to have a negative correlation in breast and prostate cell lines as well as in clinical tissues [10].

#### Post-transcriptional

Several microRNAs (miRNAs) have been identified as important post-transcriptional regulators for *RKIP* expression. miRNAs are short, non-coding RNAs that regulate gene expression post-transcriptionally [65]. They function by binding to the 3′ UTR of their target mRNAs, inhibiting their translation and the production of a mature protein. One type of miRNA that was identified to suppress *RKIP* expression is miR-27a. Li et al. [66] discovered that the upregulation of miR-27a decreased *RKIP* expression in cisplatin-resistant lung adenocarcinoma (LUAD) cell lines, which could contribute to the chemoresistance of LUAD cells to cisplatin. Another study performed luciferase assays, quantitative real-time polymerase chain reaction (qRT-PCR), and western blotting on miR-155 and noticed the downregulation of RKIP occurred at the protein rather than mRNA level, indicating probable post-translational regulation [67]. Overexpression of miR-23a was also found to decrease RKIP mRNA and protein expressions [66]. For this experiment, researchers used an RKIP 3′ UTR luciferase assay with and without mutation or deletion of miR-23a-binding site, and found that *RKIP* expression is mediated via direct binding to the miR-23a region [68]. Similarly, overexpression of miR-543 was found to downregulate *RKIP* expression in clinical prostate cancer specimens and promote the proliferation and metastasis of cancer cells [69]. Moreover, Huang et al. [70] discovered that miR-224 expression was significantly upregulated in breast cancer cell lines and enhanced metastasis.

#### Post-translational

The addition of a phosphate group to a mature RKIP protein post-translationally alters RKIP's conformation as well as its function. PKC carries out this operation when a specific signaling stimulus is present to initiate phosphorylation of RKIP at Ser153 [52]. The phosphorylation of RKIP of Ser153 inactivates the binding pocket of RKIP, thereby resulting in the release of Raf-1 and activation of MEK and ERK [52]. pRKIP has a higher affinity to GRK2, which normally acts as a negative regulator of the G protein coupled receptor (GPCR) [58, 71]. The pRKIP dimerizes GRK2 and prevents it from inhibiting the GPCR cascade, resulting in enhanced MAPK signaling [34].

### Role of RKIP in resistance

#### Resistance to chemotherapy

Cancer cells often develop therapeutic resistance to chemotherapy as well as defects in cellular death mechanisms prompting treatments to be less effective [72]. In regard to *RKIP* expression in regulating cell resistance to chemotherapy, studies have shown that RKIP functions as an apoptosis inducer, thereby causing the re-sensitization of resistant tumors to chemotherapy [73]. The transcription factor NF-κB promotes tumor resistance to chemotherapy and immunotherapy by inducing the expression of anti-apoptotic gene products related to B-cell lymphoma 2 (Bcl-2) and regulating/decreasing the expression of death receptors (DRs) [74]. Furthermore, certain small molecules including proteasome inhibitor, NPI-0052, bortezomib, and nitric oxide (NO) donor DETA/NO are able to sensitize multiple cancer cell lines to chemotherapy-related apoptosis through NF-κB and Snail inhibition and RKIP induction [31, 74–76]. A study examined the proteasome inhibitor NPI-0052 and bortezomib and discovered that both inhibited NF-κB and its downstream target, Snail (a repressor of RKIP), resulting in the derepression of *RKIP* [73]. The treatment of human prostate cancer cell lines by NPI-0052 or NO donor DETA/NO resulted in the reversal of tumor cell EMT, migration, and invasion [73, 74]. It was suggested that this occurred through RKIP-mediated NF-κB inhibition as well as the subsequent suppression of the EMT inducer, Snail [73, 74]. On the other hand, NF-κB constitutive activity has also been associated with the cause of adaptive tumor resistance to ionizing radiation [74, 77]. Studies also show that silencing Snail or RKIP ectopic induction has direct effects that suppress the expression of anti-apoptotic proteins from the Bcl-2 family. This supports the conclusion that RKIP and the NF-κB/Snail module have opposing roles in the regulation of tumor resistance.

#### Resistance to immunotherapy

Tumor resistance to conventional therapies including chemotherapy and radiation remains the major obstacle in the successful treatment of cancer patients [78]. This persistent obstacle for cancer patients has led to the development of immunotherapy with the goal to overcome resistance to drugs and radiation as well as enhancing the specificity to eliminate tumor cells [46, 78]. One approach to investigate resistance is to identify the pathways that regulate resistance and then develop interventions to disrupt these pathways in order to override resistance and sensitize resistant cells to cell death [73]. In the dysregulated NF-κB/Snail/YY1/RKIP loop, the overexpression of *NF-κB*, *Snail*, and *YY1* has led to the maintained downregulation of RKIP. The maintained hyperactivation of NF-κB and its targets, Snail and YY1, results in cell resistance and insensitivity to both chemo- and immune-therapeutic drugs [79]. In addition to RKIP inhibiting anti-proliferative and tumor suppressor functions, it also acts as an anti-resistant factor [73].

The role of RKIP in the reversal of immune resistance was examined through the RKIP disruption loop by Bonavida [73]. Both NK cells and cytotoxic T lymphocytes (CTLs) mediate their killing mechanisms by both necrotic and apoptotic mechanisms. The necrotic mechanism mediates its cell death mechanism through the perforin/granzyme system by perforating holes on the cell membrane. This perforation results in changes to the osmotic pressure, lysis of the cells, as well as apoptosis [73, 80]. On the other hand, the apoptotic mechanism begins with the interaction of ligands on the lymphocytes such as tumor necrosis factor (TNF)-α, Fas ligand (FasL), and TRAIL with the corresponding receptors (TNFR-1/2, Fas, DR4, and DR5). The contact between sensitive target cells and cytotoxic lymphocytes leads to the activation of the apoptotic pathway, thereby resulting in cell death [73].

The sensitization of Fas-resistant tumor cells to FasL cytotoxicity was achieved via the treatment of tumor cells with a NO donor that resulted in the upregulation of Fas on the tumor cells and sensitization to apoptosis. The underlying mechanism was investigated, and it was found that NO inhibited the Fas transcription repressor, YY1 [81]. In addition, upregulation of *RKIP* in tumor cells also resulted in the upregulation of Fas via RKIP-mediated inhibition of NF-κB and downstream YY1 [82]. Further studies also revealed that YY1 represses the TRAIL receptor DR5 and its inhibition by NO or by the upregulation of RKIP resulted in the sensitization of TRAIL-resistant tumor cells to TRAIL apoptosis [83, 84].

## PTEN: characteristics

### General properties

PTEN was first discovered in 1997 independently by three different laboratories [85–87]. While researchers at Duke University were studying the karyotypes of glioblastoma multiforme cell lines, they noticed that there was one copy of chromosome 10 that was lost at a much higher frequency compared to the other chromosomes [88]. Glioblastoma biopsies were compared to normal tissue DNA, and it was determined that loss of heterozygosity (LOH) for chromosome 10 was in fact frequent in glioblastoma patients [89]. Mapping studies revealed that there was a chromosomal loss in the long arm of chromosome 10 (also known as the q arm) [90]. In order to confirm whether chromosome 10 loss occurred in glioblastoma multiforme, researchers fused a normal chromosome 10 from fibroblasts with U251 glioma cell lines and noticed the formation of a tumor was suppressed [91]. The results demonstrated that chromosome 10 possesses a tumor suppressor gene that is present in glioblastoma multiforme [91].

Soon after the connection was made between chromosome 10 and glioblastoma multiforme, its relevance with other types of cancer soon followed. Studies found that PTEN was mutated not only in glioblastoma multiforme, but also prostate carcinoma [92], breast cancer [93], and endometrial carcinoma [94].

PTEN encodes a 403 amino acid protein where the amino-terminal region shares sequence homology with the actin-binding protein, tensin, and the putative tyrosine-protein phosphatase, auxilin [95]. PTEN is encoded on chromosome 10q23, a region found to express LOH in various types of cancer [96].

### PTEN expression in cancers

PTEN mutations occur in both hereditary and somatic tumor syndromes and are responsible for a large percentage of human cancers [97]. Hereditary PTEN mutations cause PTEN hamartoma tumors syndromes (PHTSs), which feature a variety of benign and malignant tumors [97]. Affected PHTS patients develop disorganized and hyperplastic cellular overgrowth, which eventually affects various tissues in the thyroid, breast, skin, and/or brain, and can also cause neurodevelopment disorders such as autism spectrum disorder [97]. In somatic cancers, including endometrial cancer (UCEC), breast cancer, prostate cancer, and glioblastoma, PTEN inactivation results in missense and nonsense mutation, mono- or bi-allelic deletion of the genomic locus or silencing through promotor methylation [98, 99].

Frequent mutations in PTEN are found in glioblastoma as almost all glioblastomas display loss of function of the PTEN tumor suppressor [100]. According to The Cancer Genome Atlas (TCGA), deletions including the PTEN locus were identified in 143 of 170 glioblastomas [100], making this the most frequent genetic change. The remaining 15% of tumors displayed reduced expression of the *PTEN* mRNA relative to sample controls [100].

Additionally, PTEN loss is a common trait in breast cancer patients as well. Multiple studies have shown through IHC analysis of patient-derived samples that PTEN protein expression is either lost or reduced in 40% of primary breast carcinomas as assessed by the IHC [100]. Reduced PTEN expression can result in homozygous deletion of the *PTEN* gene locus or epigenetic silencing of the PTEN promotor [93, 101, 102]. However, it is important to mention that the reported rates of promoter hypermethylation are diverse as some studies observe a correlation between PTEN promoter hypermethylation and breast cancer while other studies do not conclude a correlation [102, 103].

Another study by Fan et al. [104] sought to evaluate how significant the PTEN mutation is in the prognosis and drug selection of clear cell renal cell carcinoma (ccRCC), a type of kidney cancer. Their results showed that among 538 cases, 5% of patients carried the PTEN mutation including amplification, truncating, deep deletion, in-frame mutation, and missense mutations spanning across the entire gene [104]. Clinical analysis showed that patients with the PTEN mutation had poorer prognosis on survival and disease recurrence compared to patients with wt-PTEN. Furthermore, their results provide evidence that ccRCC patients with the PTEN mutation are more susceptible to distant metastasis, indicating that early intervention is necessary for patients to have a longer survival [104].

Kurose et al. [105] explored the loss of *PTEN* expression in relation to elevated phosphorylated AKT (pAKT) levels. After examining over two hundred epithelial ovarian tumors from cancer patients, Kurose et al. [105] determined that LOH data from 44 tumors were informative. Among the 44 tumor samples, 77% (34 of 44) of these tumors had either partial or complete expressional loss of PTEN at the protein level. Of the 77% of samples, 3% had structural biallelic deletion inactivation, 53% had a structural monoallelic deletion of PTEN, and the remaining 44% did not have any evidence of *PTEN* expression and, thus, might be the result of epigenetic silencing. Kurose et al. [105] concluded that there is a prominent role of PTEN inactivation in ovarian carcinomas associated with increased pAKT.

#### Crystal structure

Biochemical and biological evidence showed that PTEN is a dual lipid and protein phosphatase that removes the third inositol phosphate from the substrate phosphatidylinositol-3,4,5-bisphosphate (PIP_3_), the product in the phosphatidylinositol 3 kinase (PI3K) pathway [106]. An analysis of the crystal structure of PTEN uncovered a C2 domain that binds to phospholipids on membranes and a phosphatase domain that contains dual-specific activity toward both tyrosine (Y), serine (S)/threonine (T), and lipid substrates. As a result, PTEN is able to dephosphorylate phospho-peptides and phospho-lipids [95].

### PTEN-mediated signaling

The PTEN/AKT pathway begins with the binding of a ligand such as growth factors, cytokines, and hormones to membrane RTKs. When a ligand such as a growth factor binds to a receptor (RTKs), two RTK monomers will get close and form a dimer, which will activate the intracellular tyrosine kinase domain. In this case, dimerization will activate PI3Ks. PI3Ks are a family of lipid kinases characterized by their ability to phosphorylate the hydroxyl group of the 3rd position on the inositol ring of phosphatidylinositol [107]. PI3Ks contain two domains: one P110 and one P85 domain. PI3K activation typically occurs through the binding of the P85 subunit or through the adapter molecules such as the insulin receptor substrate (IRS) proteins [108]. If RTKs are not present, PI3K can become activated by a GTP binding to the RAS protein [108]. Activated PI3K phosphorylates phosphatidylinositol-4,5-bisphosphate (PIP_2_) to produce PIP_3_. The role of PIP_3_ is to act as a dock for phospholipids where proteins can be recruited to the plasma membrane and subsequent activation of the signaling cascade [108]. From that point, PIP_3_ can either bind directly to the AKT or PDK1 protein. If PDK1 phosphorylates the binding site Thr308 of AKT, there will be partial activation, however, phosphorylation of AKT at Ser473 will stimulate full AKT activation [108].

AKT acts as a key signaling node because it is responsible for initiating and regulating the processes of multiple downstream cytoplasmic and nuclear targets [109]. AKT is interconnected with several signaling pathways including cyclin D1 [110], glycogen synthase kinase-3B (GSK3B) [111], forkhead [112], and BAD [113]. Therefore, pAKT is responsible for modulating processes involving cell survival, progression, DNA repair, angiogenesis, and cellular migration [109]. Overexpression of this signaling pathway results in abnormal cell proliferation (oncogenesis) [114].

**Figure 2.**
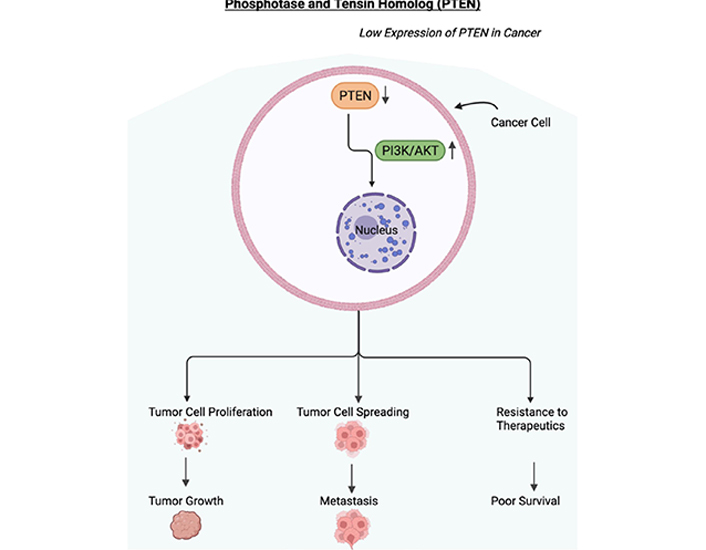
Low expression of *PTEN* in cancer. Schematic diagram depicting the downregulation or loss of expression of *PTEN*. In cancer cells, PTEN has been shown to be downregulated, mutated, or lost resulting in the upregulation of the PI3/AKT pathway. The activated PI3/AKT pathway will be involved in the proliferation of tumor cells, the spreading of tumor cells, and resistance to therapeutics. These will result in tumor growth, metastasis, resistance to therapeutics, and in poor survival of the cancer patient. Figure created with BioRender.com

PTEN dephosphorylates the lipid substrate PIP_3_ at the 3′ position converting PIP_3_ back to PIP_2_, thereby halting the phosphatidylinositol 3 (PI3)/AKT mitogenic signaling pathway. PTEN acts as the central negative regulator of PI3K by opposing its activity and dephosphorylating PIP_2_ into PIP_3_. A lack of PTEN leads to elevated levels of pAKT (Figure 2) [115].

### Regulation of *PTEN* expression

#### Transcriptional

Several transcription factors have been shown to positively and negatively affect the transcription of PTEN. Positive regulators of PTEN include the early growth response protein 1 (EGR-1), tumor protein 53 (p53), active transcription factor 2 (ATF2), and peroxisome proliferator-activated receptor gamma (PPARγ) [116, 117]. PPARγ belongs to a family of nuclear receptors that is responsible for lipid and glucose metabolism regulation and improving endothelial function [115]. Though other nuclear receptors generally will bind to a single specific ligand, PPARγ has the ability to bind to numerous natural ligands, especially fatty acids [118]. These transcription factors were shown to directly bind to the promoter region of PTEN thereby regulating its transcription [116].

Virolle et al. [119] found that EGR-1 binds to the PTEN 5′ UTR containing a functional GCGGGGGCG EGR-1 binding site. In addition, they discovered that inducing EGR-1 by exposing cells to ultraviolet light upregulated the expression of PTEN mRNA and protein, thereby leading to apoptosis [119]. Similarly, p53 upregulates PTEN transcription by binding to the functional p53 binding element upstream of the PTEN promoter [120]. To determine ATF's association with PTEN, researchers used ChIP assays to see if ATF2 would bind to both PTEN's binding sites in the promoter region *in vivo* site 1: (cctTGACGggtggg) and site 2: (ggcTGACGgccatt). What researchers noticed was ATF2 bound to both sites in the PTEN promoter, however, the basal binding of ATF2 to site 2 was higher than site 1 [117]. Furthermore, Patel et al. [121] discovered that activation of PPARγ by selective ligand upregulated *PTEN* expression in human macrophages. To test their experiment, Patel et al. [121] used antisense oligonucleotides (AS) to abolish the increased expression of PPARγ during monocyte differentiation over a seven-day time period. Their data, supported by western blot analysis, demonstrated a selective loss of PPARγ protein as well as a > 95% reduction in PTEN mRNA and protein content by day seven [121].

On the other hand, NF-κB negatively regulates *PTEN* expression. To confirm whether the downregulation of PTEN by NF-κB occurs at the transcriptional or post-translational level, Vasudevan et al. [9] co-transfected NIH 3T3 cells with luciferase reporter assay to determine the effect of p65 on the promoter of PTEN. P65 is one of the two (p50 and p65) heterodimeric transcription activators for NF-κB. Their results demonstrated that p65 repressed the PTEN promoter and that NF-κB activation was sufficient enough to inhibit *PTEN* expression [9].

Other transcription factors that were reported to inhibit *PTEN* expression in several cancer models were: mitogen-activated protein kinase kinase-4 (MKK4) and B-lymphoma Moloney murine leukemia virus (Mo-MLV) insertion region 1 (BMI1) [116]. To determine the effect of MKK4 on PTEN, Xia et al. [122] used genetic approaches to modulate the expression of *MKK4* in mouse embryo fibroblast (MEF) cells and non-small cell lung cancer (NSCLC) cells. Their findings demonstrated that in both MEF and NSCLC cells, high *MKK4* expression correlated with low *PTEN* expression [122]. Similarly, high *PTEN* expression was associated with a reduction in intracellular phosphoinositides, which are required for AKT activation [122]. They found that MKK4 was responsible for inhibiting *PTEN* expression by nuclear translocation of p65 and activation of NF-κB [122]. Moreover, the relationship between BMI1 and PTEN was observed. Studies showed that when BMI1 binds to PTEN exclusively in the nucleus, nuclear PTEN negatively regulates *BMI1* expression through its C-terminal domain [123].

#### Epigenetic

Furthermore, lysine-specific demethylase 1 (LSD1), EZH2, and G9a were reported to epigenetically regulate *PTEN* expression. In a study by Yokoyama et al. [124], ChIP assay was carried out using Neuro2a cells. The immunoprecipitated chromatin samples were subject to quantitative polymerase chain reaction (qPCR) using primers corresponding to the indicated promoter regions of the *PTEN* gene. They used the distal region of the PTEN promoter as a negative control (NC). However, after inspection they noticed that LSD1 was in the promoter region (not the distal region) of PTEN, suggesting that nuclear LSD1 (nLSD1) targets PTEN. Afterward, ChIP analysis was used to confirm if the nLSD1 complex modifies histones at the PTEN promoter. Knockdown of Myt1 by siRNA decreased the amount of LSD1 recruitment at the PTEN promoter, which indicated that nLSD1 directly binds to the PTEN promoter through Myt1. To further validate their results, they also noticed that Neuro2a cell proliferation decreased as a result of LSD1 or Myt1 expression, which makes sense considering how PTEN negatively regulates cell proliferation.

Similarly, in another study, Yang et al. [125] analyzed expression levels of *EZH2* as well as LSD1 on osteosarcoma cells. Their data revealed that downregulated EZH2 and LSD1 could dramatically increase the expression level of *PTEN* [125]. Yang et al. [125] next carried out a ChIP assay to confirm their results, and the data showed that EZH2 and LSD1 can directly bind to the promoter regions of PTEN where it demethylates H3K27me3.

A study by Bhat et al. [126] identified PTEN to be a direct target of G9a. By performing Nanostring PanCancer pathway analysis they were able to identify the PI3K signaling pathway as a target downstream of G9a. Next, they decided to focus on PTEN, a well-known regulator of the PI3K/AKT pathway. They discovered that PTEN mRNA was significantly upregulated in G9a knockdown cells, indicating that PTEN mRNA is regulated by G9a in a methylation-dependent manner [126]. After they used ChIP-seq, they discovered that G9a enrichment was evident at the PTEN promoter. The loss of *PTEN* expression by G9a resulted in reduced AKT activity and consequent proliferation [126].

#### Post-transcriptional

Likewise, there is a plethora of miRNAs that have been found to bind to the 3′ UTR of PTEN mRNA. These miRNAs include miR-21, miR-22, miR-214, miR-205, miR-552, miR-106b, and miR-93. Meng et al. [127] evaluated the expression levels of miR-21 in hepatocellular cancer (HCC) cells by expression profiling and discovered that when miR-21 was inhibited, *PTEN* expression increased. In contrast, when the miR-21 expression was enhanced, there was increased tumor cell proliferation, migration, and invasion [127]. In another study, researchers measured PTEN abundance by IHC and identified a highly significant inverse correlation between the abundance of PTEN and miR-22 [128]. They also found a direct correlation between the miR-22 and pAKT [129]. Jindra et al. [129] identified that miR-214 was significantly upregulated after T cell activation. To test their hypothesis, they performed stem-loop Taqman^©^ real-time polymerase chain reaction (RT-PCR) assays and noticed that their data were consistent with their hypothesis. To further characterize the role of miR-214, Jindra et al. [129] then isolated CD4^+^ and CD8^+^ T cells. They discovered that at 72 h following stimulation, levels of PTEN mRNA were substantially reduced in both CD4^+^ and CD8^+^ cells while displaying an increase in miR-214 expression [129]. Moreover, Li et al. [130] discovered the miR-205 directly inhibited PTEN by performing a dual-luciferase reporter assay. In their study, they inserted the wt or mutated 3′ UTR of PTEN mRNA downstream of the luciferase reporter gene along with LV-miR-205. Their results showed that miR-205 inhibited the luciferase reporter activity of wt PTEN 3′ UTR, but the inhibition was less changed for 3′ UTR with mutated binding sites [130]. Furthermore, Zhao et al. [131] used RT-PCR and western blot analysis to detect the expression of miR-552 and PTEN, respectively. They detected that PTEN mRNA was downregulated in miR-552 overexpression ovarian cancer (OV) cells, and there was a significant negative correlation between miR-552 and *PTEN* mRNA expressions in human OC tissues [131]. On a similar note, expression levels of miR-106b and miR-93 were detected by Li et al. [132] by immunofluorescence (IF) staining. Their studies revealed upregulation of miR-106b and miR-93 in MCF-7 cells reduced *PTEN* expression. However, downregulation of miR-106b and miR-93 in MDA-MB-231 cells increased *PTEN* expression, all of which indicate how *PTEN* expression is inversely related to miR-106b and miR-93 [132]. Their data suggest that the effect of miR-106b and miR-93 on migration, invasion, and proliferation of breast cancer can be reversed by *PTEN* expression [132].

On the other hand, there is increasing evidence of long noncoding RNAs (lncRNAs) regulating *PTEN* expression and the progression of human cancers. One type of lncRNA that was found to regulate *PTEN* expression in liver cancer stem cells was lnc-DILC [133]. lnc-DILC is located at the chromosomal locus 13p24 and acts as a tumor suppressor for tumorigenesis and metastasis in liver and colorectal cancer. In their study, Zhang et al. [133] collected 68 pairs of ccRCC and normal tissue samples from patients at the Luoyang Central Hospital. qRT-PCR assays showed that lnc-DILC expression was remarkably decreased in ccRCC tissues in comparison to normal tissues, and a decrease in lnc-DILC expression was correlated with a larger tumor size [133]. In order to identify possible candidate proteins that interact with lnc-DILC, Zhang et al. [133] then performed RNA pull-down assay and mass spectrum analysis. They discovered that PTEN mRNA levels were not affected by knockdown or overexpression of lnc-DILC; however, PTEN protein level was significantly increased after lnc-DILC overexpression. Therefore, it was concluded that lnc-DILC regulated PTEN expression at the post-translational level [133].

In addition, another study by Xin et al. [134] discovered that in patients with liver cancer, lnc-HULC was negatively correlated with *PTEN* expression. In their study, Xin et al. [134] obtained thirty cases of liver cancer tissues from patients who underwent surgery. *In situ* hybridization (ISH) and RT-PCR conveyed that HULC expression was significantly increased in liver cancer tissues compared to noncancerous tissues. To analyze PTEN expression, IHC staining and western blotting were then performed. They found a significant reduction in PTEN protein expression in liver cancer tissues in comparison to noncancerous tissues. Taken all together, it was concluded that at the translational level, there is a strong negative correlation between lnc-HULC and PTEN [134].

Furthermore, a study by Wang et al. [135] explored the relationship between lnc-GAN1 and NSCLC. In their study, human NSCLC specimens were obtained from 194 patients at Sun Yat-Sen University Cancer Center. After using qRT-PCR, researchers identified that lnc-GAN1 expression was significantly decreased in comparison to normal lung tissue samples. Patients with low lnc-GAN1 expression had a significantly poorer overall survival (OS) and disease-free survival (DFS) compared to those with high lnc-GAN1 expression. Using qRT-PCR, Wang et al. [135] discovered that *PTEN* expression was downregulated in NSCLC tissues and positively correlated with lnc-GAN1 expression. Their results concluded that lnc-GAN1 upregulates and activates PTEN mRNA and protein levels [135].

Moreover, another study by Yang et al. [136] investigated another lncRNA, lnc-FTX, in cardiac hypertrophy of neonatal mouse cardiomyocytes induced by angiotensin II (Ang II). In their study, 95% of their mouse cells were stained using IF for alpha-smooth muscle actin (α-SMA), in order to determine the presence of cardiac myocytes. The expression of lnc-FTX was higher in the control groups compared to the groups treated with Ang II, signifying that lnc-FTX plays a role in mouse cardiac myocyte hypertrophy. Next, Yang et al. [136] evaluated the expression of *PTEN* in the PI3/AKT signaling pathway. Their results showed that *PTEN* expression induced by Ang II was increased by lnc-FTX, though PI3K/AKT expression induced by Ang II was reduced by lnc-FTX. Ultimately, lnc-FTX reduced mouse cardiac myocyte hypertrophy (when induced by Ang II) by aiding in the release of PTEN and inhibiting the PI3/AKT signaling pathway [136].

On another note, Yan et al. [137] investigated lnc-MIR17HG and its role in acute myeloid leukemia (AML, also known as LAML used for indicating the TCGA database). In their study, forty patients with AML had enrolled to participate in their study. To determine the effect of lnc-MIR17HG on the PTEN mRNA and protein expression levels, researchers used qRT-PCR and western blot analyses. They discovered that lnc-MIR17HG overexpression significantly upregulated PTEN mRNA and protein expression levels in comparison to the control groups: untransfected cells and cells transfected with NC mimics [137]. In addition, the results showed that lnc-MIR17HG mRNA expression level was decreased in AML, supporting a potential tumor-suppressive role, and regulation of PTEN [137].

#### Post translational

Furthermore, there are various enzymes responsible for phosphorylation of PTEN on the C-terminal domain including casein kinase 2 (CK2), GSK3, and Rho-associated (RhoA) protein kinase (ROCK).

CK2 directly phosphorylates PTEN at specific C-terminal seryl and threonyl residues namely Ser370, Ser380, Ser385, Thr382, and Thr383 [138]. In one study, Torres and Pulido [138] used polymerase chain reaction (PCR) oligonucleotide site-directed mutagenesis to analyze *PTEN* expression. They found that mutations of residues at Ser370 and Ser385 significantly reduced CK2-induced phosphorylation and PTEN phosphorylation *in vivo*. On the other hand, mutations of Ser380, Thr382, and Thr383 had a smaller effect [138]. They concluded that mutations of Ser370, Ser380, Ser385, Thr382, and Thr383 resulted in protein stability. Similarly, in another study, Miller et al. [139] used mass spectrometric methods to identify *in vivo* phosphorylation sites of PTEN. They discovered CK2 phosphorylates Ser370 and Ser385 to high stoichiometry *in vitro* while Thr366 was also phosphorylated, but to a lower extent [139].

GSK3 is a serine/threonine protein kinase that was originally identified to have functions in the regulation of glycogen synthase, but was later found to have roles in biochemical processes. GSK3 is sometimes regarded as a moonlighting protein because of the multiple processes it controls [140]. GSK3 phosphorylates serine or threonine residues, though it has been reported that there is a modestly higher activity against serine [141]. Al-Khouri et al. [142] discovered that by using phosphoamino acid analysis, GSK3β phosphorylated PTEN at Ser362 and Thr366. In order to confirm whether GSK3β participates in PTEN phosphorylation in intact cells, Al-Khouri et al. [142] reduced the cellular levels of GSK3β and GSK3α by RNA interference, and then immunoprecipitated PTEN. Afterward, they probed it with an anti-phosphothreonine-proline antibody, and they noticed that in cells with reduced levels of both GSK3 isoforms, phosphorylation of PTEN at Thr366 was much reduced. This concluded that GSK3 does indeed phosphorylate Thr366 in intact cells.

ROCK is a well-known effector of the small GTPase, RhoA, that upregulates the activity of PTEN. Yang and Kim [143] examined AKT phosphorylation levels in response to changes in RhoA or ROCK activity. What they noticed was that when RhoA activity was inhibited by the transfection of cells with siRNA, AKT phosphorylation increased. However, the transfection of active RhoA by PMT treatment had downregulated AKT phosphorylation. Similarly, inhibition of ROCK activity by siRNA did increase AKT phosphorylation. These results demonstrated that activated RhoA-ROCK downregulates AKT phosphorylation [143]. In this experiment, Yang and Kim [143] wanted to investigate whether PTEN is a downstream effector of RhoA-ROCK that regulates AKT phosphorylation. One way to test this is by looking at PTEN activity, specifically regarding its expression and phosphorylation levels. So, what researchers found was that PTEN activity was downregulated after ROCK activity was inhibited. Moreover, the downregulation of PTEN activity upregulated AKT phosphorylation [143].

### Role of PTEN in resistance

#### Resistance to chemotherapy

The effects of combining two GSK3β inhibitors, namely 9-ING-41 and 9-ING-87, with chemotherapy were examined on breast cancer cells. In a study by Ugolkov et al. [144], researchers discovered that the inhibition of GSK3 by the two small molecule inhibitors suppressed the growth of breast cancer cells but had little effect on non-tumorigenic cell growth. In their study, 9-ING-41 potentiated the effect of the chemotherapeutic drug irinotecan CPT-11 *in vivo*, thereby leading to the decrease of BC-1 and BC-2 tumors in mice [144]. Altogether, their results suggest that GSK3 is a promising therapeutic approach to overcome chemoresistance in human breast cancer [144]. This relates to PTEN because GSK3β can mediate the phosphorylation of AKT and PTEN to promote cell migration and apoptosis, which may promote chemoresistance in breast cancer [145]. Additionally, the mediation of GSK3β can regulate cell viability through the PTEN/PI3K/AKT signaling pathway to promote migration and apoptosis [145].

The modulation of *PTEN* expression or mutation in many cancers resulted in the activation of the PI3K/AKT pathway that regulates tumor cell proliferation, invasion, and resistance to cytotoxic drugs [146, 147]. Several reports have demonstrated that the inactivation of PTEN contributed to tumor cells unresponsiveness to cytotoxic chemotherapeutic drugs. Several examples are illustrated briefly. Fang et al. [148] have reported that the expression level of miRNA-17-5p was elevated in patients who were resistant to chemotherapy. They found that PTEN was a target of miRNA-17-5p in colon cancer cells and this interaction was responsible for the multi-drug resistant phenotype. In addition, the treatment of cells with chemotherapy increased the expression of miRNA-17-5p that further repressed PTEN and the development of chemo-resistance. Jian et al. [149] reported the role of miRNA-193-3p in human gastric cancer. They found that miRNA-193-3p was upregulated in both gastric cancer cell lines and human gastric tumors. The downregulation of miRNA-193-3p inhibited tumor cell proliferation, migration, and the resistance to 5-fluorouracil (5-FU) both *in vitro* and *in vivo* [149]. PTEN was a target of miRNA-193-3p and responsible for the chemoresistance. In a similar study, Jin et al. [150] reported that miRNA-141-3p was overexpressed in resistant esophageal cancer cells from patients. Inhibition of miRNA-141-3p reversed the resistance and the cells underwent apoptosis to 5-FU and oxaliplatin. PTEN was a target of miRNA-141-3p. There was also an inverse relationship between the expression of *PTEN* and miRNA-141-3p in cancer tissues. *In vivo* inhibition of miRNA-141-3p mouse models resulted in the inhibition of tumor growth [150].

Liao et al. [151] reported on the resistance of glioma to temozolomide (TMZ) which is a standard drug for glioma. They found that lncRNA cancer susceptibility candidate 2 (CASC2) expression was downregulated in tumor tissues and correlated with poor survival time. Overexpression of CASC2 sensitized the tumor cells to TMZ. In addition, CASC2 upregulated PTEN protein and inhibited pAKT protein. The upregulation of PTEN by CASC2 was via the direct inhibition of miRNA-181 [148]. Li et al. [152] reported that lncARSR was upregulated in HCC and associated with large tumor size and poor prognosis. The overexpression of lncARSR augmented the resistance of HCC cells to doxorubicin (DOX) *in vitro* and *in vivo* [152]. They noted that lncARSR physically interacts with PTEN mRNA and promoted PTEN degradation leading to the activation of the PI3K/AKT pathway. They suggested that lncARSR may serve as a prognostic biomarker and therapeutic target [152]. Vahabi et al. [153] reported that miR-96-5p targets *PTEN* expression and affected the chemosensitivity and radiosensitivity of head and neck squamous cell carcinoma (HNSCC) cells. Also, miR-96-5p activated the PI3K/AKT/mammalian target of rapamycin (mTOR) pathway. They also suggested here that miR-96-5p could serve as a biomarker for chemo-radio sensitivity [150]. Ding et al. [154] reported that miR-21 interaction with PTEN regulated the sensitivity of LUAD cells to 5-FU-mediated cytotoxicity. Overexpression of miR-21 inhibited 5-FU-mediated apoptosis in cancer cells [154]. Wu et al. [155] reported that overexpression of *PTEN* resulted in the induction of apoptosis (intrinsic mitochondrial pathway) in breast cancer cells and also inhibited cell proliferation. The overexpression of *PTEN* also resulted in reversing the chemoresistance [155]. Hence, they suggested that PTEN is a potential target for chemotherapeutics [152]. Dou and Zhang [156] reported that PTEN was a direct target miR-4461 in OV. There was an inverse correlation between the expression of *PTEN* and miR-4461 in OV tissues. In addition, miR-4461 was in part responsible for the resistance of OV cells to cisplatin [156]. Fischer et al. [157] reported that PTEN-deficient tumors are addicted to the DNA damage kinase ataxia telangiectasia mutated kinase (ATM) in order to detect and repair induced DNA damage [157]. The use of low concentration of ATM inhibitor synergized with radiation to treat PTEN-deficient tumors in radiation-resistant lung cancer models [157].

#### Resistance to immunotherapy

Immune checkpoint blockade (ICB) is an important and increasingly popular approach in immunotherapy while many cancers still remain insensitive to ICB [140]. GSK3 inhibitors, including GSK3i and SB415286, were observed to downregulate transcription of programmed cell death protein 1 (PD-1) in CD8^+^ T cells [158]. Suppression of GSK3 was effective in preventing the growth of the B16 melanoma or the El4 lymphoma *in vivo* tumor studies [158]. The conditional genetic deletion of GSK3α and β in mice displayed reduced PD-1 expression on CD8^+^ T cells and suppressed B16 primary metastasis to the same degree as deficient PD-1 [158]. The transfer of T cells treated with a GSK3 inhibitor delayed the growth of EL4 lymphoma [158]. These results demonstrate that GSK3 inhibitors, namely GSK3α and β, that downregulate PD-1 expression can enhance CD8^+^ T cells function to a similar extent to PD-1 blocking antibodies, which offer novel approaches in cancer immunotherapy [158]. This connects to PTEN because GSK3β can mediate the phosphorylation of AKT and PTEN to promote chemoresistance [159]. Identifying GSK3 inhibitors, such as GSK3i and SB415286, are important because they not only inhibit the PTEN/PI3K/AKT signaling pathway, but they also downregulate transcription of PD-1 in CD8^+^ T cells, which activates CD8 cytotoxic T cells and decreases tumor proliferation, invasion, and cell cycle progression [160].

In another study by Peng et al. [161], researchers evaluated the impact PTEN suppression has on T cell meditated anti-tumor responses. In their study, they used western blotting to analyze decreased PTEN and increased pAKT expression in A375 melanoma cells. They discovered that by decreasing *PTEN* expression, the percentage of lysed tumor cells significantly reduced when the cells were co-cultured with the pmel-1 T cells *in vitro*. To determine the *in vivo* effects of PTEN suppression on T cell-mediated anti-tumor activity, they established an adoptive T cell therapy (ACT) murine model and found that the accumulation of tumor-reactive T cells in A375 melanoma tumors had significantly reduced [161]. They concluded that loss of PTEN can cause resistance to T cell-mediated immune responses and resistance to immunotherapy in melanoma [161].

Peng et al. [161] also wondered whether inhibiting the PI3K pathway would improve the effectiveness of immunotherapy. While some of the targets of the PI3K pathway are critical to cell function and viability, the PI3Kβ isoform can regulate AKT activity in tumors with PTEN loss [161]. As a result, they tested whether selectively inhibiting PI3Kβ would increase the efficacy of immunotherapy in melanomas [161]. They used a PI3Kβ inhibitor, GSK2636771, and discovered that it reduced the activation of the AKT pathway and moderately (< 20%) inhibited the growth of three human melanoma cells while PTEN was absent [158]. They examined the role of PI3Kβ on T cell-induced apoptosis and found that the small molecule inhibitor, GSK2636771, improved the T cell-induced tumor thereby killing all the melanoma cell lines [161]. Peng et al. [161] concluded that the PI3Kβ inhibitor enhanced the efficacy of immunotherapy in melanomas with PTEN loss.

Additionally, a study by Chida et al. [162] sought to analyze predictors of response to PD-1 blockade among patients with microsatellite instability-high (MSI-H)/mismatch repair-deficient (dMMR) tumors [162]. In their study, forty-five patients with MSI-H/dMMR Gi tumors, including but not limited to, gastric, colorectal, and pancreatic cancer (PAAD) were analyzed with having PD-1 blockade. Using transcriptomic analysis and multiplex fluorescence IHC, the tumor microenvironment (TME) was evaluated. They discovered that in common oncogenic signaling pathways, the only mutation that was correlated with significantly low objective response rates (ORRs) after PD-1 blockade was PTEN. PTEN mutations in the phosphatase domain were also associated with significantly shorter progression-free survival (PFS) and OS compared to wt PTEN. However, this was not true for PTEN mutations in the C2 domain. PTEN mutations in the phosphatase domain also exhibited fewer CD8^+^ T cells and increased tumor-associated macrophages in immunosuppressive TME. In addition, PTEN-mutated tumors were highly associated with low levels of PTEN mRNA and the loss of PTEN protein, which resulted in the advancement of the PI3K/AKT signaling pathway. Their results concluded that PTEN mutations in the phosphatase domain are correlated with PTEN loss of function, resulting in resistance to PD-1 blockade [162].

Furthermore, another study by Lin et al. [163] discovered that mRNA delivery by polymeric nanoparticles (NPs) can effectively induce the expression of *PTEN* when it is mutated in melanoma cells and lost in prostate cancer cells. In this study, Lin et al. [163] developed a polymeric NP platform for PTEN mRNA to deliver to several PTEN-null or mutated tumor samples. Their NP results showed that these PTEN mRNA cells not only restored the susceptibility of tumor cells to death but also led to the release of damage-associated molecular patterns (DAMPs). Additionally, they found that mRNA delivery induces the activation of autophagy, which could promote additional DAMPs from secreting [163]. *In vivo* results further exhibited that PTEN restoration induced powerful CD8^+^ T cells responses and also reversed the immunosuppressive microenvironment [163]. In fact, they found that the combination of PTEN mRNA NP with an immune checkpoint inhibitor (ICI) antibody [anti-programmed cell death ligand 1 (PDL1)] results in a highly potent anti-tumor effect when observed in a subcutaneous mutated PTEN model from melanoma and PTEN-null prostate cancer model [163]. Their findings suggested that mRNA nanomedicines that restore tumor suppressors may provide a potent treatment for a variety of malignancies in the future [163].

One study by Agrawal et al. [164], observed dendritic cell (DC) levels in elderly (65–90 years of age) as well as young subjects (20–35 years of age), and found that the PTEN levels in DCs were higher in elderly subjects compared to young subjects. As a result of higher PTEN levels, there was reduced AKT activation, antigen uptake, and DC migration [161]. Interestingly, their studies revealed the activation of the p65 subunit of NF-κB at the basal level and after activation with DNA was significantly higher (*P* < 0.05) in DCs from elderly subjects compared to young subjects [164]. The phosphorylation of the p65 subunit was also significantly greater in the DCs from aged subjects compared to young subjects [164]. Their data suggest that the increased levels of NF-κB activation in DCs from elderly subjects can account for the increased PTEN expression as well as increased reactivity to human DNA [164].

## Cross-talk signaling pathways between RKIP and PTEN

### Indirect regulation between MAPK and PI3/AKT pathway

One reason we believe there exists an indirect regulation between RKIP and PTEN is because B-RAF mutations are involved in the dysregulation of the MAPK and PI3K/AKT pathways. B-RAF mutations are present in up to 70% of melanoma cases [165]. Therefore, it is important to determine if these signaling crosstalk pathways are related indirectly and/or directly as they can offer novel targets for therapeutic interventions. In fact, the most promising treatments for melanoma seem to target mutations involved in the RAF-MEK and PI3K pathways. B-RAF is a member of the RAF family proteins that are involved in the signal transduction cascade involved in the regulation of apoptosis, proliferation, and transformation to cancerous states [166]. B-RAF provides the most signal in the transduction pathway because of its elevated basal kinase activity that is not present in either A-RAF or C-RAF. Additionally, both A-RAF and C-RAF require phosphorylation and additional activation by tyrosine kinase, Src, to induce hyperactivation. However, given the fact that phosphorylation is the only requirement for B-RAF activation, and aspartic acid provides B-RAF with a greater propensity towards activation, it is no surprise that the majority of activating mutations occur in B-RAF.

**Figure 3.**
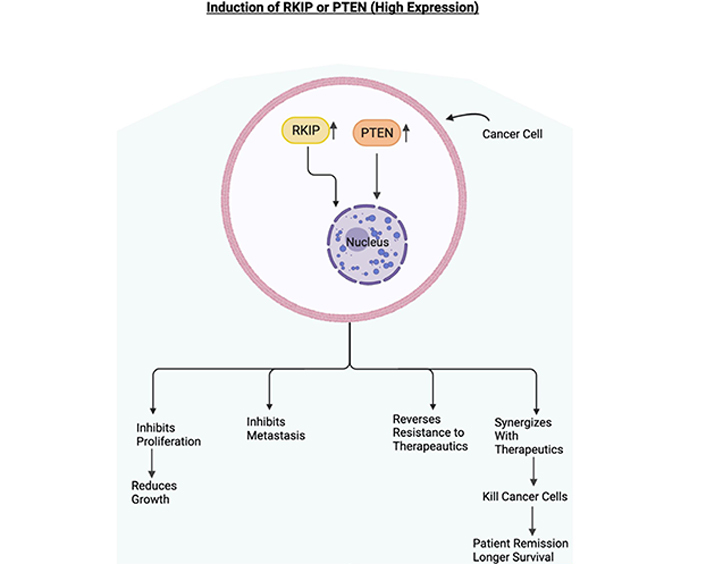
Role of RKIP and PTEN in cancer. Schematic diagram depicting the overexpression of RKIP and PTEN. In cancer cells, if RKIP and PTEN are overexpressed, they will be mediating the inhibition of cell proliferation, the inhibition of metastasis, sensitization to cytotoxic therapeutics. The sensitization to therapeutics will lead to the killing of cancer cells, which will eventually lead to the patient's remission and longer survival. Figure created with BioRender.com

As shown in this review, RKIP negatively regulates the MAPK pathway via the inhibition of B-RAF activity. The inhibition of B-RAF by RKIP results in a downregulation of all metastatic pathways associated with B-RAF (Figure 3). Similarly, in the PI3K/AKT pathway, AKT, and more specifically the isoform AKT3, plays a role in regulating B-RAF, which promotes cell proliferation and metastasis [165]. In a study by Tran et al. [167], researchers wanted to identify whether AKT3 would phosphorylate mutant B-RAF to determine whether this process played any role in melanoma development. Using a combination of pharmacological and genetic approaches, they found that AKT3 downregulated B-RAF to promote tumor progression [167]. AKT3 directly phosphorylated B-RAF on residues Ser364 and Ser428, thereby promoting rather than inhibiting, melanocytic cell growth [167]. PTEN is involved in this pathway because deletion of PTEN results in the hyperactivation of PI3K/AKT pathway and an overexpression of AKT3 [165].

### Indirect regulation via NF-κB/Snail/YY1 loop

Similarly, we believe there exists another indirect relationship between RKIP and PTEN via the dysregulated NF-κB/Snail/YY1 loop expressed in cancer [5]. As mentioned earlier, the transcription factor NF-κB binds with the inhibitory protein IκB to keep it in its inactive form. However, IκB has the potential to become phosphorylated by active IKK, which results in the activation of NF-κB. When NF-κB is activated, it translocates into the nucleus and interferes with the transcription of many genes. In cancer cells, NF-κB activity results in the inhibition of antitumor immunity, tumor cell proliferation, and angiogenesis. Therefore, RKIP's role in inhibiting NF-κB is paramount in controlling the proliferation of cancer cells [5].

NF-κB inhibition results in the downstream inhibition of putative metastasis inducers YY1 and Snail. Snail is transcriptionally regulated by NF-κB and YYI, but it inhibits RKIP. In the majority of cancer cells, *Snail* is overexpressed in part as a result of NF-κB hyperactivation and *YY1* overexpression. When *Snail* is overexpressed, it inhibits RKIP. In turn, RKIP fails to inhibit the NF-κB pathway. In the majority of human cancer tissues, RKIP was shown to be downregulated, partially as a result of NF-κB hyperactivation and Snail. NF-κB hyperactivation is maintained when RKIP is downregulated and *YY1* is overexpressed. However, when *RKIP* is overexpressed, it inhibits NF-κB and its downstream targets, YY1 and Snail [60].

**Figure 4.**
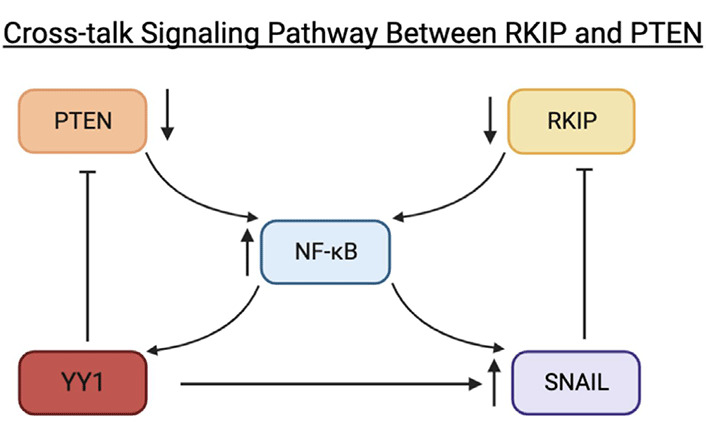
Schematic diagram depicting cross-talk signaling pathways between RKIP and PTEN via the NF-κB/Snail/YY1 loop. In the dysregulated loop, NF-κB becomes activated by active IKK. Active NF-κB will activate downstream Snail and YY1. The activation of Snail, a repressor of RKIP, will result in the inhibition of RKIP. As a result, RKIP inhibition will maintain NF-κB hyperactivation. When NF-κB becomes hyperactivated, *YY1* becomes overexpressed and, as a repressor of PTEN, will result in the inhibition of PTEN. Figure created with BioRender.com

We believe there exists another indirect relationship between RKIP and PTEN because *PTEN* expression is low in the majority of cancers, in part by NF-κB and YY1. PTEN is either downregulated or absent in most cancer cells because NF-κB hyperactivation and overexpression of *YY1* (PTEN inhibitor) decrease *PTEN* expression [5, 168]. When PTEN is inhibited, the AKT/PI3K pathway becomes activated and results in tumor cell survival, growth, and resistance [5]. Essentially, the hyperactivation of NF-κB and YY1 results in the downstream inhibition of RKIP in the MAPK pathway and PTEN in the PI3K/AKT. Therefore we believe in the dysregulated loop, there exists a direct relationship between both gene products or pathways (Figure 4).

## Bioinformatic analyses

In the last two decades, great efforts have been done to fully characterize the molecular profile of tumors through the development of novel and high-sensitive molecular techniques [169, 170]. The molecular profiling of tumors has generated a huge amount of bioinformatics data whose interpretation is particularly challenging due to some limitations mainly represented by the different sources of data and the need for sophisticated algorithms for the statistical interpretation and elaboration of records [171]. To effectively cope with these limitations, International Consortia and independent researchers have developed several databases and portals for the easy interpretation of cancer computational data, of these the most widely used are TCGA, which collects molecular data on several patients affected by 33 different tumors, and Gene Expression Profile Interacting Analysis (GEPIA), which allows researchers worldwide to analyze and interpret the molecular data contained in the TCGA database [172, 173]. By using both GEPIA and the molecular data contained on TCGA, bioinformatics analyses exploring the direct and indirect molecular interactions existing between *RKIP* and *PTEN* gene products were performed in order to establish their involvement in the development of different tumors as well as their potential prognostic value in cancer.

First, dysregulation in the expression levels of both *PTEN* and *RKIP* was investigated in both tumor and normal samples of 31 different tumors by using GEPIA.

**Figure 5.**
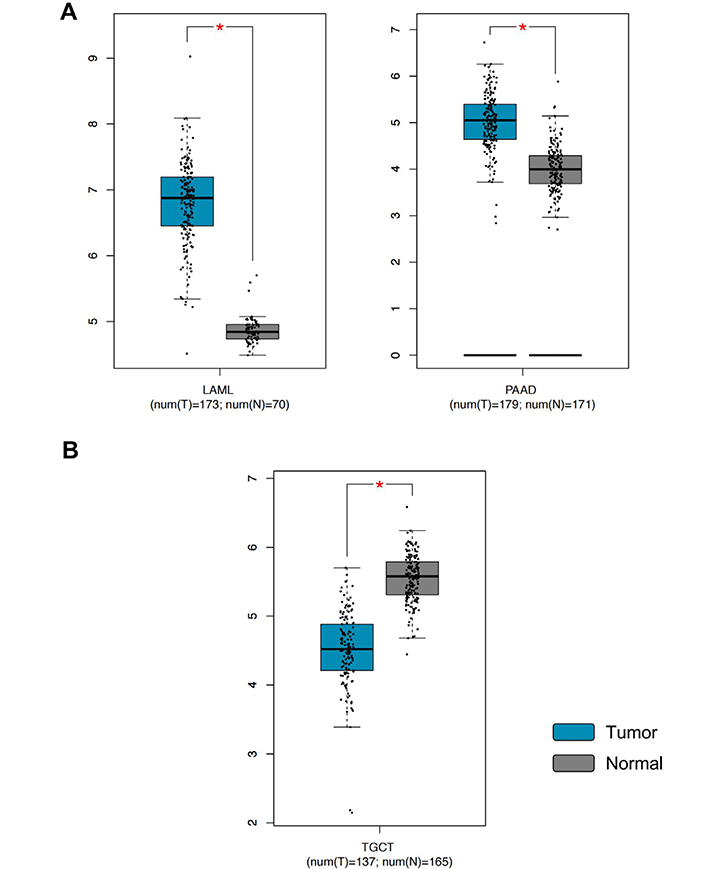
*PTEN* gene expression levels in LAML, PAAD, and TGCT samples compared to the controls. A) *PTEN* overexpression in tumors; B) PTEN down-regulation in tumors. ^*^ *P* < 0.01, *P* values adjusted according to the Benjamini and Hochberg false discovery rate. The relative expression levels were first log2(TPM+1) transformed and the log2FC was defined as median (tumor)—median (normal), where TPM is the transcript count per million. FC: fold change; TPM: transcripts per million

GEPIA analysis revealed that PTEN was significantly dysregulated in three out of 31 tumors investigated. More in detail, *PTEN* expression levels were significantly upregulated in LAML and PAAD, while *PTEN* expression levels were significantly down-regulated in testicular cancer (TGCT) compared to the normal testis (Figure 5).

**Figure 6.**
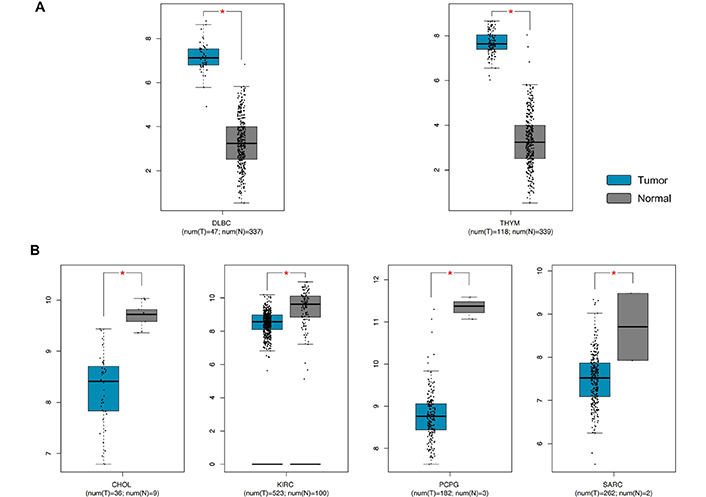
*RKIP* gene expression levels in DLBC, THYM, CHOL, KIRC, PCPG, and SARC samples compared to the controls. A) *RKIP* overexpression in tumors; B) RKIP down-regulation in tumors. ^*^ *P* < 0.01, *P* values adjusted according to the Benjamini and Hochberg false discovery rate. The relative expression levels were first log2(TPM+1) transformed and the log2FC was defined as median (tumor)—median (normal), where TPM is the transcript count per million

The same analysis performed for RKIP expression levels demonstrated a significant dysregulation of RKIP in six out of 31 tumors analyzed. In this case, RKIP levels were significantly increased in large diffuse B-cell lymphoma (DLBC) and thymoma (THYM), while significant down-regulation was observed in bile duct cancer (CHOL), kidney clear cell carcinoma (KIRC), pheochromocytoma and paraganglioma (PCPG) and sarcoma (SARC) (Figure 6).

These data suggest how both PTEN and RKIP are significantly dysregulated in different tumors. More in detail, *PTEN* overexpression observed in LAML samples is controversial as different studies demonstrated that PTEN acts as a tumor suppressor in myeloid leukemia and its expression levels are lower in LAML samples compared to controls [174, 175]. Similarly, also *PTEN* overexpression observed in PAAD samples is in contrast with different data. In this case, it was demonstrated as PTEN suppression is associated with PAAD aggressiveness and a worse prognosis, therefore, it is reasonable to assume that as a tumor suppressor, PTEN levels should be decreased in PAAD samples compared to normal ones [176, 177]. On the contrary, the PTEN down-regulation observed in TGCT samples was already reported in literature where it was demonstrated that its suppression is associated with the transition from intratubular germ cell tumors to invasive germ cell tumors [178].

No specific studies about the overexpression of *RKIP* in DLBC and THYM have been performed except for another computational study performed by Zaravinos and colleagues [74] which analyzed the same data here and investigated obtaining very similar results. Contrariwise, it was demonstrated as RKIP down-regulation is strongly associated with cholangiocarcinoma cell metastasis [179]. In line with our results, other studies revealed that the loss of *RKIP* expression is associated with poor outcomes in SARC patients thus representing a good prognostic indicator for these patients [180] as well as with the risk of development of ccRCC [181]. No data about pheochromocytoma are still available.

**Figure 7.**
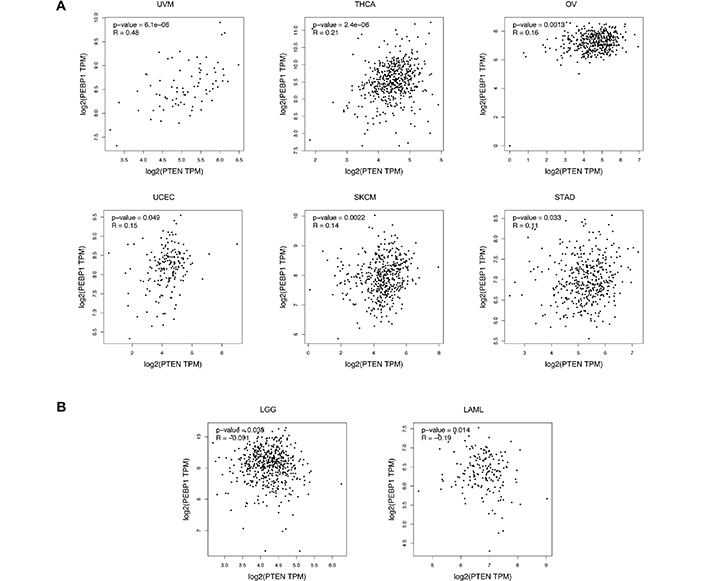
GEPIA correlation analyses between the expression levels of *PTEN* and *RKIP* in different tumors. A) RKIP-PTEN positive correlation; B) RKIP-PTEN negative correlation. Pearson's correlation test was adopted, and data were considered statistically significant for *P* < 0.05. The relative expression levels were first log2(TPM+1) transformed and the log2FC was defined as median (tumor)—median (normal), where TPM is the transcript count per million

To further investigate the molecular relationship existing between the two genes, correlation analyses between *PTEN* and *RKIP* expression levels were performed in different tumors. This second approach revealed that PTEN and RKIP are positively or negatively correlated in a significant manner in 8 of 33 different tumors. In particular, positive correlations were observed in six different tumors, including uveal melanoma (UVM), thyroid cancer (THCA), OV, UCEC, skin cutaneous melanoma (SKCM), and stomach cancer (STAD), while negative correlations were observed for low-grade glioma (LGG) and LAML (Figure 7).

Overall, the results obtained demonstrated the existence of weak positive or negative correlations existing between *RKIP* and *PTEN* expression levels in tumor samples, except for UVM where a moderate and positive correlation (R = 0.48) was observed. Noteworthy, both UVM and SKCM showed positive correlations, suggesting how in these two similar tumors the alteration of the cross-talk existing between RKIP and PTEN could drive neoplastic transformation.

**Figure 8.**
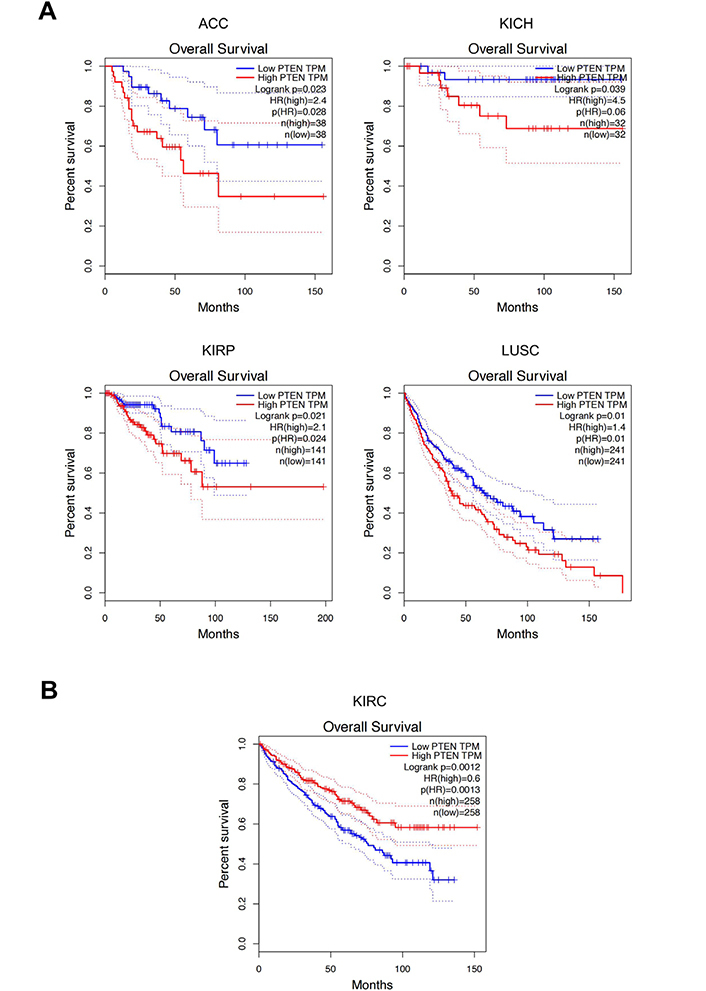
Survival analyses according to *PTEN* expression levels performed by GEPIA. A) *PTEN* overexpression is associated with poor patients' OS; B) *PTEN* low expression levels are associated with poor patients' OS. *P* values were calculated by using the logrank test. Data were considered statistically significant for *P* < 0.05. HR: hazard ratio

To further clarify the prognostic role of both PTEN and RKIP, further bioinformatics analyses were performed by analyzing the expression and survival data contained in the TCGA database. GEPIA analyses of *PTEN* expression and survival data highlighted a good prognostic value of PTEN for five different tumors, i.e. adrenocortical cancer (ACC), kidney chromophobe cancer (KICH), kidney papillary cell carcinoma (KIRP), lung squamous cell carcinoma (LUSC), and KIRC. In particular, higher expression levels of *PTEN* were associated with a worse prognosis in all cancer except KIRC where high *PTEN* expression was correlated with a good prognosis (Figure 8).

**Figure 9.**
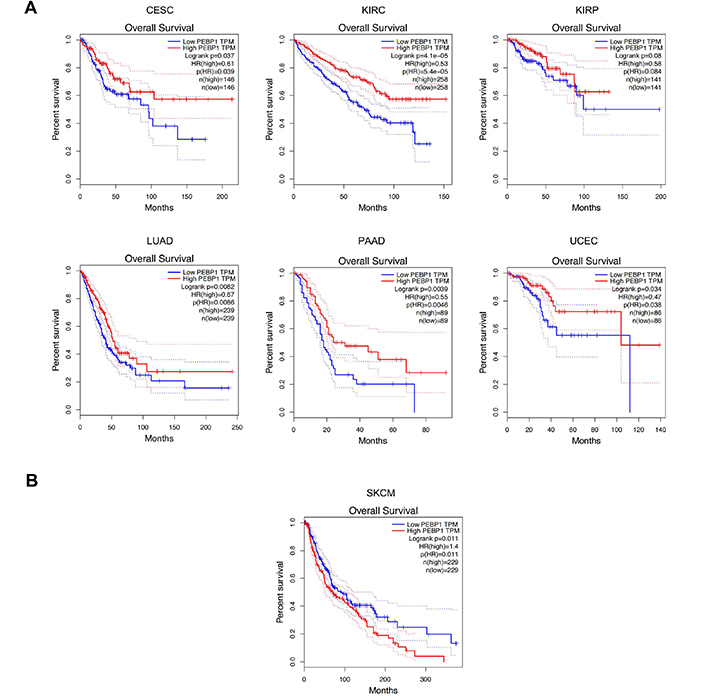
Survival analyses according to *RKIP* expression levels performed by GEPIA. A) *RKIP* overexpression is associated with poor patients' OS; B) *RKIP* low expression is associated with poor patients' OS. *P* values were calculated by using the logrank test. Data were considered statistically significant for *P* < 0.05

Similarly, the same analyses performed for RKIP expression levels demonstrated a greater prognostic value of RKIP compared to PTEN as it is associated with the prognosis of seven different tumors. In detail, low expression levels of RKIP were significantly associated with poorer survival in cervical cancer (CESC), KIRC, KIRP, LUAD, PAAD, and UCEC (Figure 9A), while high RKIP expression levels are associated with a poor prognosis in SKCM patients (Figure 9B).

All these data suggest that the concomitant evaluation of both PTEN and RKIP expression levels could give important information to predict the aggressiveness of the disease and the prognosis of patients. Noteworthy, the analyses here performed demonstrated that both PTEN and RKIP can be considered useful biomarkers in kidney cancers as both genes are associated with patients' prognosis when dysregulated. Such results are in line with other published studies which demonstrated that both RKIP and PTEN are key determinants of patients' prognosis and response to therapies [104, 181–183].

## Discussion

In this review, we have examined several of the shared properties mediated by the tumor suppressors RKIP and PTEN and their roles in the regulation of many phenotypic properties of cancer cells. Both *RKIP* and *PTEN* expression levels are downregulated in various cancers that result in several manifestations such as cell viability, proliferation, invasion, metastasis, and resistance to both chemo- and immune-therapeutics. The low expression of *RKIP* participates in the activation of the NF-κB pathway and the induction of downstream targets that are involved in many of the above-mentioned tumor phenotypic activities. Likewise, the downregulation of PTEN also plays a role in the activation of the PI3K/AKT/mTOR pathway that regulates similar tumor phenotypic activities. The shared anti-tumor activities of RKIP and PTEN are the result also of their cross-regulation, directly and indirectly, via cross-signaling pathways. Bioinformatic analyses confirmed their direct relationship in many human cancers.

Analyses of the signaling pathways that RKIP regulates intracellularly consisted of inhibiting the Raf/MEK/ERK and the NF-κB pathways [27, 49]. Through PKC-mediated phosphorylation of Ser153, RKIP is no longer able to bind and inhibit the RAF and NF-κB pathways though the phospho-RKIP binds and inhibits GRK2 which enhances its signaling [54]. The downregulation of RKIP in many cancers is regulated at multiple levels and depending on the cancer type studied.

RKIP is regulated positively and negatively by various transcription factors. For instance, transcriptionally, RKIP is positively regulated by Sp1, CREBB, and p300 acetylase protein [56]. In addition, the AR transcriptionally regulates *RKIP* expression [50, 57]. RKIP is negatively regulated by Snail and EZH2 [10, 59]. Likewise, BACH1 is a transcription repressor of RKIP [61]. Epigenetically, RKIP is negatively regulated by EZH2 and post-transcriptionally RKIP is negatively regulated by various miRNAs described in the text. RKIP in the cytosol is also regulated by PKC that phosphorylates RKIP on Ser153 and inhibits its binding activity to RAF while it regulated GRK2 [31, 73]. As discussed, RKIP is involved in the regulation of tumor cells responses to chemo and immunotherapy; its downregulation in cancers results in the activation and upregulation of resistant factors [30, 73]. Hence, RKIP induction reverses the resistance phenotype by inhibiting the resistance factors. PTEN shares many similarities with *RKIP* activities and its expression in cancers is altered via downregulation, mutation, or deletion. This leads to the activation of the PI3K/AKT pathway and downstream the induction of target genes involved in proliferation, invasion, and resistance [144]. It also is involved in the cross-talk signaling with the MAPK and NF-κB pathways. *PTEN* expression is regulated positively and negatively by various factors. Positively, PTEN is regulated by EGR-1, p53, ATF2, and PPARγ [117]. Negatively, PTEN is regulated by the NF-κB pathway, by MKK4 and BMI1 [117]. Epigenetically, LSD1, EZH2, and G9a regulate *PTEN* expression [123, 125, 126]. Also, post-transcriptionally, several miRNAs have been reported to regulate *PTEN* expression as discussed in this report. Post-translationally, phosphorylation of PTEN by CK2, GSK3, and ROCK was reported and discussed herein [138, 142, 143]. PTEN, like RKIP, is also involved in the regulation of resistance. Its altered expression in many cancers coincided with resistance to cytotoxic drugs [146].

Based on the above findings, it is clear that there exists signaling cross-talks between RKIP and PTEN, namely, the cross-talk between the MAPK and the PI3K/AKT pathway and the dysregulated NF-κB/Snail/YY1/RKIP/PTEN in cancers whereby the downregulation/inhibition of either RKIP or PTEN results in a reciprocal regulation of the other gene product. Thus, it will be expected that there will be a direct correlation between the expressions of *RKIP* and *PTEN* in several cancers. This was analyzed by bioinformatics in 31 different human tumors and it was revealed that there was a positive correlation in 6 different tumors and a negative correlation in 2 tumors. In the positive correlations, it confirms the existence of signaling cross-talks between RKIP and PTEN. It also suggests that targeting either RKIP or PTEN for induction will result in targeting the other as well and will inhibit the tumor phenotype and reverse resistance in combination with current therapeutics.

Furthermore, the effect of hypoxia on PTEN and RKIP and their interrelationship has been an emerging area of interest. Hypoxia is a state in which tumor cells are not provided with sufficient amounts of oxygen. As a result, this fuels the progression of solid tumors from tumor plasticity, genomic instability, and heterogeneity by metabolically reprogramming the TME to worsen the poorly vascularized conditions [184]. Hypoxia downregulates DNA repair processes including homologous recombination, non-homologous end joining (NHEJ), and mismatch repair [184]. The downregulation of these repair pathways contributes to genetic heterogeneity in tumors including chromosomal instability, point mutations, and genome-doubling events [185]. Interestingly, DNA structural changes including large deletions, copy number aberrations, duplications, and truncations are more frequent than single nucleotide alterations [186]. Hypoxia is associated with increased mutational load across cancer types and occurs in key driver genes such as *TP53* and *PTEN* [184]. In a study by Bhandari et al. [186], the investigators observed a significant interaction between hypoxia and altered PTEN. In this study, they showed that the PTEN mRNA is regulated by both PTEN mutational status as well as hypoxia. Moreover, they observed that PTEN mRNA levels were lowest when PTEN was altered and there was elevated hypoxia [186].

The concept of targeting hypoxia-induced pathways as a model for cancer therapy is established [187]. Hypoxia creates a physical barrier conducive to tumor survival, which in the context of TME promotes an immunosuppressed microenvironment that can be resistant to ICI [184]. Some studies support the potential for therapy involving immunotherapy and hypoxia-based therapy [184]. However, there is some difficulty in these interventions because hypoxia-induced factors (HIFs) inhibitors have poor selectivity, so therapies will often inhibit downstream pathways or include the use of hypoxia-activated prodrugs [187]. Some studies have found that the inhibition of HIF transcription factors can be achieved by targeting the PI3K/AKT/mTOR pathways [188–190]. An earlier report found a possible interrelationship between tuberous sclerosis complex 2 (TSC2), which is a molecular regulator of mTOR, and HIF-1α (a subunit of HIF-1). In their study, they found that TSC2 knockout resulted in increased HIF-1α accumulation and upregulation of HIF-induced genes including vascular endothelial growth factor (*VEGF*) [191].

Another study by Zhong et al. [191] found that the expression of HIF-1α was blocked by LY294002 and rapamycin. This is important for PTEN as well as RKIP because LY294002 is an inhibitor of PI3K [191]. In their study, *HIF-1*-dependent gene transcription was blocked by dominant-negative AKT or PI3K and by wt PTEN. However, transcription was stimulated by active AKT or dominant-negative PTEN [191]. Even better, Karar et al. [192] investigated NVP-BEZ235 and found that it was more potent than both rapamycin and LY294002 by blocking HIF-1α induction by decreasing protein translation and increasing cell killing under hypoxia, likely by increasing apoptosis.

Yang et al. [193] examined whether PI3K/AKT and the MEK/ERK signaling pathways are involved in regulating the expression of *HIF-1α* and *VEGF*. VEGF is a substance that stimulates the formation of new blood cells and is one of the most well-known angiogenic factors in choroidal neovascularization (CNV). Yang et al. [193] discovered that PI3K/AKT was needed for the expression of *HIF-1α* and *VEGF*, however, MEK/ERK was needed for the expression of *VEGF*. Yang et al. [193] did mention that they cannot exclude the effect of MEK/ERK on HIF-1α activation under hypoxia because hypoxia does promote ERK phosphorylation and translocation to the nucleus. These findings relate to the role of PTEN in the negative regulation of the PI3K/AKT pathway and its indirect role in the regulation of hypoxia.

Currently, there are no agents that directly target the induction of RKIP or PTEN. For RKIP induction, however, several reports using various agents resulted in the upregulation of RKIP and the inhibition of tumor growth and the reversal of resistance. Among these agents NO donors, treatment of tumor cells with low expression of *RKIP* and resistant to cytotoxic drugs resulted in the induction of RKIP and the sensitization to both chemo- and immune-therapeutics. NO inhibits NF-κB and downstream the Snail repressor of RKIP as well as the YY1 activator of Snail [81, 82]. The inhibition of NF-κB and other hyperactivated signaling pathways in cancer cells inhibit several anti-apoptotic gene products and activate several pro-apoptotic gene products that sensitized the cells to apoptosis by chemotherapeutic drugs [5, 9, 10]. In addition, the inhibition of the immune suppressor YY1 transcription factor resulted in the upregulation of the apoptotic receptors, Fas and DR5, on the tumor cells making them sensitive to apoptosis by the CTL and NK bearing the corresponding ligands, FasL and TRAIL, respectively [73, 80]. Also, treatments with siRNA for Snail or YY1 resulted in similar findings as above [31, 81]. Clearly, based on the regulation of *RKIP* expression discussed in the text, there are several potential factors that can be targeted specifically including Snail and BACH1.

Studies by Baritaki et al. [84] reported that treatment with the proteasome inhibitor, NPI-0052 resulted in the inhibition of EMT and Snail and induction of RKIP. The resistant tumor cells were sensitized to chemo-immunotherapeutics.

Zaravinos et al. [74] reviewed the role of RKIP as a therapeutic target. Briefly, various agents that induce the interaction of RKIP with its partners include rituximab, dihydroartemesinin, and didymin all of which upregulate *RKIP* expression. Also, drugs such as oxaliplatin and camptothecin inhibit the inactive RKIP phosphorylation.

Trastuzumab (anti-Her2 mmAB) is a targeted cancer drug widely known to treat early and advanced breast and STAD expressing Her2 [184]. However, the overall response rate of trastuzumab is low given that less than 35% of patients with overexpressed ErbB2 metastatic breast cancer respond to trastuzumab [194–196]. Therefore, in an effort to identify trastuzumab resistance-conferring, Nagata et al. [197] investigated whether PTEN activation is involved in the rapid AKT dephosphorylation before PI3K inhibition and ErbB2 downregulation. Nagata et al. [197] used untreated and trastuzumab-treated SKBr3 cells after immunoprecipitation to examine PTEN activity. They discovered that trastuzumab-treated cells dramatically increased PTEN activity after 20 min concluding that the rapid increase of PTEN activity could account for the rapid AKT dephosphorylation before ErbB2 is downregulated and PI3K is inhibited [197].

In an effort to determine the anti-tumor functions of DOX and resveratrol (RES) on gastric cancer and its treatment, Xu et al. [198] used the cell counting kit-8 (CCK8) assay to detect the cytotoxicity of DOX and RES to gastric cancer cells and the protein expressions of the PTEN/AKT signaling pathways. Their results demonstrated that DOX treatment combined with RES significantly activated *PTEN* expression and inhibited AKT phosphorylation. Their study verified that RES inhibited the AKT signaling pathway through the upregulation of PTEN. The inhibition of the AKT signaling pathway subsequently led to EMT inhibition, suppression of cell migration, and reversal of DOX-resistant cells. Shi et al. [199] investigated the effects of miR-29a in chemo-resistant colon cancer cells. They investigated the human colon cancer cell line HT29 and DOX-resistant colon cancer HT29/DOX cells. Their results revealed that miR-29a inhibition resulted in the upregulation of PTEN in HT29/DOX cells and resulted in the reversal of resistance by inhibiting cell proliferation and the induction of apoptosis through the PI3K/AKT pathway. Another study by Lin et al. [163] demonstrated that mRNA delivery by polymeric NPs effectively induces *PTEN* expression to induce autophagy and trigger cell death. Lin et al. [163] systemically delivered PTEN mRNA to prostate cancer tumors. This delivery markedly inhibited the growth of human prostate cancer cells both *in vitro* and *in vivo*. Furthermore, PTEN reactivation improved the sensitivity of PTEN tumors to ICB therapy as well as diminished the immunosuppressive tumor environment. Several miRNAs have been reported to reverse *PTEN* expression whether to activate or inactivate it. One miRNA, namely miR-205, was examined by Li et al. [200] to investigate its effects on reversing DOX resistance through the upregulation of PTEN. The inhibition of miR-205p led to the overexpression of *PTEN* while the overexpression of miR-205 led to downregulation of PTEN. As a result, the inhibition of miR-205 plays a key role in reversing drug resistance in liver cancer cells through enhancing apoptosis via PTEN upregulation.

## Abbreviations

5-FU: 5-fluorouracil
AML: acute myeloid leukemia
Ang II: angiotensin II
ARs: androgen receptors
ATF2: active transcription factor 2
BACH1: BTB and CNC homology protein 1
BMI1: B-lymphoma Moloney murine leukemia virus insertion region 1
CASC2: cancer susceptibility candidate 2
ccRCC: clear cell renal cell carcinoma
ChIP: chromatin immunoprecipitation
CK2: casein kinase 2
CREB: cyclic adenosine monophosphate response element binding protein
DC: dendritic cell
DLBC: large diffuse B-cell lymphoma
DOX: doxorubicin
DRs4: death receptors 4
EGR-1: early growth response protein 1
EMT: epithelial-mesenchymal transition
ERK: extracellular signal-regulated kinase
EZH2: enhancer of zeste homolog 2
FasL: Fas ligand
GDP: guanosine diphosphate
GEPIA: Gene Expression Profile Interacting Analysis
GRK2: G protein coupled-receptor kinase-2
GSK3B: glycogen synthase kinase-3B
GTP: guanosine triphosphate
HCC: hepatocellular cancer
HIFs: hypoxia-induced factors
ICB: immune checkpoint blockade
IHC: immunohistochemistry
IKK: IkappaB kinase
IκB: IkappaB
KIRC: kidney clear cell carcinoma
KIRP: kidney papillary cell carcinoma
lncRNAs: long noncoding RNAs
LOH: loss of heterozygosity
LSD1: lysine-specific demethylase 1
LUAD: lung adenocarcinoma
MAPK: mitogen-activated protein kinase
miRNAs: microRNAs
MKK4: mitogen-activated protein kinase kinase-4
mRNA: messenger RNA
mTOR: mammalian target of rapamycin
NF-κB: nuclear factor-kappaB
NK: natural killer
nLSD1: nuclear lysine-specific demethylase 1
NO: nitric oxide
NPs: nanoparticles
NSCLC: non-small-cell lung cancer
OS: overall survival
OV: ovarian cancer
p53: protein 53
PAAD: pancreatic cancer
pAKT: phosphorylated AKT
PCPG: pheochromocytoma and paraganglioma
PD-1: programmed cell death protein 1
PEBP2: phosphatidylethanolamine binding protein 2
PI3: phosphatidylinositol 3
PI3K: phosphatidylinositol 3 kinase
PIP_2_: phosphatidylinositol-4,5-bisphosphate
PIP_3_: phosphatidylinositol-3,4,5-bisphosphate
PKC: protein kinase C
PPARγ: peroxisome proliferator-activated receptor gamma
pRKIP: phosphorylated Raf kinase inhibitor protein
PTEN: phosphatase and tensin homolog
qRT-PCR: quantitative real-time polymerase chain reaction
RES: resveratrol
RhoA: Rho-associated
RKIP: Raf kinase inhibitor protein
ROCK: Rho-associated protein kinase
RTKs: receptor tyrosine kinases
RT-PCR: real-time polymerase chain reaction
SARC: sarcoma
siRNA: small interfering RNA
SKCM: skin cutaneous melanoma
Sp1: specificity protein 1
STAD: stomach cancer
TCGA: The Cancer Genome Atlas
TGCT: testicular cancer
THYM: thymoma
TME: tumor microenvironment
TSS: transcription start site
UCEC: endometrial cancer
UTR: untranslated region
UVM: uveal melanoma
VEGF: vascular endothelial growth factor
wt: wild-type
YY1: Yin Yang 1

## Declarations

### Availability of data and materials

The data reported in the manuscript are available from the corresponding author.

### Copyright

© The Author(s) 2023.
